# Advancements in Microbial Applications for Sustainable Food Production

**DOI:** 10.3390/foods14193427

**Published:** 2025-10-05

**Authors:** Alane Beatriz Vermelho, Verônica da Silva Cardoso, Levy Tenório Sousa Domingos, Ingrid Teixeira Akamine, Bright Amenu, Bernard Kwaku Osei, Athayde Neves Junior

**Affiliations:** Bioinovar-Biotechnology Center, Institute of Microbiology Paulo de Góes, Federal University of Rio de Janeiro (UFRJ), Rio de Janeiro 21941-902, Brazil; verocardoso@micro.ufrj.br (V.d.S.C.); levydomingos@yahoo.com.br (L.T.S.D.); ingrid.akamine@micro.ufrj.br (I.T.A.); amenubright@micro.ufr.br (B.A.); bernardosei49@yahoo.com (B.K.O.)

**Keywords:** food additives, food preservation, fermentation, microorganisms, contamination control

## Abstract

This review consolidates recent advancements in microbial biotechnology for sustainable food systems. It focuses on the fermentation processes used in this sector, emphasizing precision fermentation as a source of innovation for alternative proteins, fermented foods, and applications of microorganisms and microbial bioproducts in the food industry. Additionally, it explores food preservation strategies and methods for controlling microbial contamination. These biotechnological approaches are increasingly replacing synthetic additives, contributing to enhanced food safety, nutritional functionality, and product shelf stability. Examples include bacteriocins from lactic acid bacteria, biodegradable microbial pigments, and exopolysaccharide-based biopolymers, such as pullulan and xanthan gum, which are used in edible coatings and films. A comprehensive literature search was conducted across Scopus, PubMed, ScienceDirect, and Google Scholar, covering publications from 2014 to 2025. A structured Boolean search strategy was applied, targeting core concepts in microbial fermentation, bio-based food additives, and contamination control. The initial search retrieved 5677 articles, from which 370 studies were ultimately selected after applying criteria such as duplication removal, relevance to food systems, full-text accessibility, and scientific quality. This review highlights microbial biotransformation as a route to minimize reliance on synthetic inputs, valorize agri-food byproducts, and support circular bioeconomy principles. It also discusses emerging antimicrobial delivery systems and regulatory challenges. Overall, microbial innovations offer viable and scalable pathways for enhancing food system resilience, functionality, and environmental stewardship.

## 1. Introduction

The increasing global population and climate change pose significant challenges for the sustainable food system [1,2]. Microbiology has been increasing its involvement in the food industry, as microorganisms and their bioproducts offer sustainable and eco-friendly solutions for this sector. Examples include biocontrol agents [3], biofertilizers/stimulants [4,5], food additives [6], enzymes [7], proteins [8], and colorants [9], among others [8]. Microbial enzymes have multiple applications. Enzyme-producing microorganisms can be isolated from food waste. Cellulolytic bacteria were isolated from fruit waste, indicating that this raw material is a source of cellulose-degrading bacteria [10]. Alkaline proteases, for instance, can hydrolyze proteins in muscle fibers and connective tissue, and they also promote protein decolorization in blood [11,12]. An amylase from a *Bacillus* sp. was isolated from agro-waste rice polish and used as a substrate [13]. The microorganism cells and parts of cellular structures represent ingredients and additives in the form of probiotics and postbiotics, respectively [14]. Polymers, such as beta-glucans, can be used as prebiotics, adding functional properties to foods [15]. Chitosan and alginate are polysaccharides widely used for soil enhancement, improving water retention, and enhancing nutrient availability [16]. These bioproducts and their functions are commonly utilized in the food industry.

Microorganisms possess various characteristics, making them a viable alternative source of products for the food industry. Bacteria, mycelium-forming fungi, unicellular yeasts, and microalgae are the primary groups involved in industrial applications. An advantage of using microorganisms and their metabolites is their low carbon footprint, as well as additional benefits such as independence from cultivable land, no climate dependency, and cultivation in bioreactors under controlled physicochemical conditions, ensuring excellent reproducibility. This culture system in bioreactors requires only small-scale environments for efficient production [17]. Additionally, the possibility of genetic modifications through synthetic biology enhances the production of products of interest [18,19].

In addition to all microorganisms and microbial products used directly in the food industry, the fermentation process is a traditional and ancient method for producing fermented foods and beverages from certain natural raw materials. It is an essential component of the diet in many societies, providing benefits for human health [20,21]. For the growth of microorganisms, it is possible to utilize agro-waste as a nutrient source in culture media. The transformation of waste into valuable resources strengthens the connection to the circular economy, thereby adding economic, sustainability, and environmental benefits to the process [19,22,23]. Another example is the transformation of banana stem waste into cellulose nitrate, a potential precursor for bioplastics [22].

The number of start-ups and companies focused on microbial-derived foods is increasing globally due to the versatility of these applications [23,24]. Microbial biomass, derived from bacteria, yeasts, filamentous fungi, or microalgae, is a promising alternative to traditional food and feed sources [25].

The focuses of the review were: (i) microbial bioproducts used in the food sector; (ii) fermentation process and its products; (iii) microbial food additives and ingredients such as organic acids, texturizers and stabilizers, colorants, sweeteners, and flavoring and aroma compounds. Additionally, the review will address (iv) probiotics, prebiotics, and postbiotics, as well as (i) methodologies for evaluating contamination and food preservation.

## 2. Literature Search Strategy

Scopus, PubMed, ScienceDirect, and Google Scholar (2014–2025) were searched using Boolean strings covering three clusters: (i) microbial fermentation, (ii) microbial bioproducts, and (iii) food safety and contamination control.

### 2.1. Eligibility Criteria

Peer-reviewed articles or reviews in English (2014–2025) that addressed microbial applications in food systems were included. Conference abstracts, theses, non-full-text works, and Duplicate entries across databases were excluded

### 2.2. Screening and Selection Process

**Figure 1:**
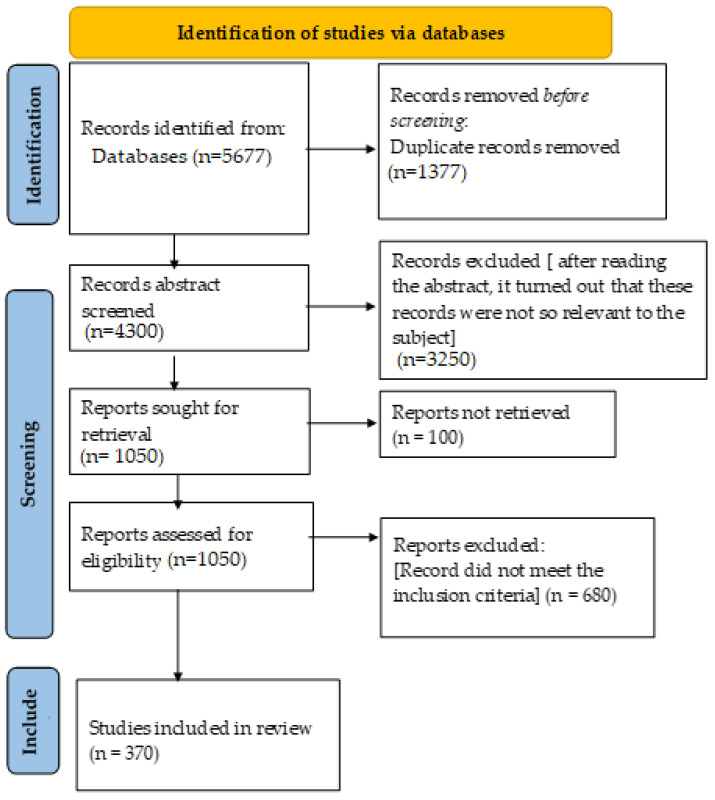
PRISMA flow diagram for the narrative review, which included searches of databases (2014–2025).

The search retrieved 5677 records, and 1377 duplicates were removed. A total of 4300 unique articles were screened based on their titles and abstracts. A total of 1050 full-text articles were assessed, and 370 studies were ultimately included. Screening was performed independently by the authors’ reviewers, with discrepancies resolved by consensus. This selection process reduced selection bias. A PRISMA-style diagram summarizes the workflow (Figure 1).

### 2.3. Data Extraction and Synthesis

From each reference, we extracted microbial taxa, process/compound type, food system application, and reported outcomes. Thematic domains narratively synthesized results. No meta-analysis was performed.

## 3. Fermentation

Fermentation is an ancient food technology that dates back to around 7000 BC. Since the dawn of civilization, methods for processing raw materials have led to the fermentation of milk, meat, and vegetables, resulting in the development of foods that are not only preserved but also delicious [24,26]. Fermentation is a chemical process that transforms organic matter through microbial metabolism facilitated by enzymes [27].

Modern industrial biotechnology is based on fermentation, and its applications are expanding across various sectors. It has traditionally been used in the food industry, and it is now evolving with the help of omics sciences, high-throughput and combinatorial library screening, synthetic biology, enzyme engineering, informatics, molecular cloning, and other innovative methods. These tools enhance microbial workhorse strains, optimize metabolic pathways, improve product yields, and facilitate downstream bioprocess scale-up, enabling industrial-scale production of food industry ingredients [28,29]. Furthermore, recent advancements in omics technologies, including metagenomics, metabolomics, and transcriptomics, have improved our understanding of the genetic, functional, and metabolic characteristics of microbial communities involved in fermentation processes [30].

One of the most advanced applications of engineered microbes in today’s food ecosystem is the production of ingredients and additives. In the past, microorganisms were selected by random mutagenesis and selection. However, they are currently modified by genetic and metabolic engineering in precision fermentation (PF) to produce high-value functional food ingredients [31]. In the food industry, various types of fermentation are employed. Each type is selected based on the objective and desired characteristics of the final product.

### 3.1. Controlled Fermentation Process

Submerged Fermentation (SmF) and Solid-State Fermentation (SSF) are both controlled fermentation processes, although the level of control varies significantly between them. PF can be applied in both SmF and, to a limited extent, SSF. Controlled fermentation is preferred in regulated sectors (pharma, biotech, infant foods) due to the strict microbial control and safety standards. Controlled fermentation processes offer a high degree of safety and consistency due to the use of well-characterized, often GRAS (Generally Recognized as Safe) microbial strains, sterile conditions, and tightly monitored parameters such as pH, temperature, and aeration. These systems are governed by strict regulatory frameworks, particularly when applied to pharmaceuticals or food-grade products. Comprehensive safety evaluations—including tests for sterility, toxin absence, and batch traceability are mandatory to ensure product safety and reproducibility [32,33].

#### 3.1.1. SmF

It occurs in a liquid medium and is widely used to produce enzymes, organic acids, fermented beverages (including dairy and alcoholic products), yogurt, and food condiments such as vinegar [34].

**Figure 2:**
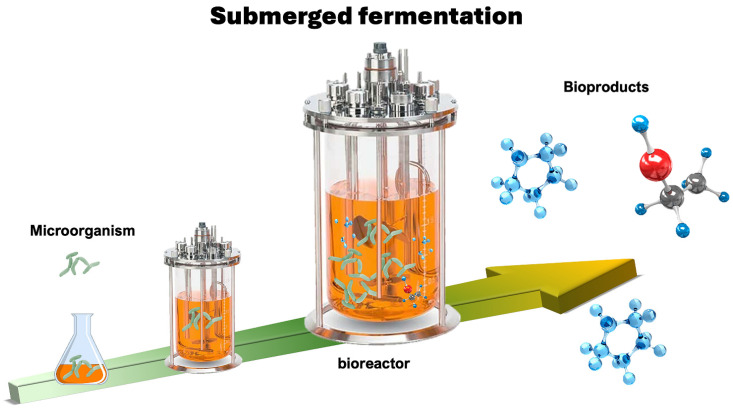
SmF for the production of a bioproduct. The selected microorganism, which produces bioproducts, is added to the optimized culture medium. These products can either be secreted directly into the supernatant, facilitating separation in the upstream process, or, if they are cellular, they will need to be isolated. An example of an extracellular bioproduct is a beta-glucan from fungi, and an example of a cellular bioproduct is an intracellular enzyme.

This fermentation exhibits advantages in stability and control parameters, including temperature, pH, nutrient distribution, and oxygen transfer. The control of these parameters and the system configuration is crucial for processing viability and industrial scale-up, and it is easier to establish than SSF. Levan, a polysaccharide, has antioxidant, anti-inflammatory, and anti-diabetic benefits. It is a versatile ingredient in the food industry, and it has been found to have applications as an emulsifier, functional prebiotic, and natural sweetener. The yeast *Saccharomyces kilbournensis* is a levan producer in SmF using sugarcane molasses [35,36]. Figure 2 is a representative scheme of SmF.

The fermentation of protein-rich food substrates with probiotics is a source of bioactive peptides. Bacteria, fungi, and yeasts can be utilized in fermentation; however, the use of probiotics has increased in recent years due to their beneficial properties, including those of probiotics such as *Lactobacillus*, *Bacillus*, and *Bifidobacterium*. *Pediococcus* genus secretes proteolytic enzymes that are essential for breaking down proteins into smaller peptides during SmF, which provide various positive health effects [37]. Another example is the antihypertensive peptides produced by *L. helveticus* in milk and dairy products [38].

#### 3.1.2. SSF

SSF uses solid substrates without excess free water. A classic example is the production of fermented foods, such as miso, Tempeh, and soy sauce, as well as the biotransformation of agro-industrial waste. Enzymes, flavors, and other compounds are also produced during this fermentation process. Lipases [39], xylanases [40], enoglucanases [40], and amylases and peptidases [41] are typical examples.

Essential features of SSF in sustainable food bioprocessing include low water and energy usage. Low water reduces energy consumption for sterilization, cooling, and wastewater treatment. Another advantage is the use of food or agricultural waste in fermentation [42]. There are innovative methods of controlling pH, aeration, agitation, pressure, temperature, and flow control [43]. Some methods include novel bioreactor designs, infrared sensors, thermocouples [44], and the addition of calcium chloride to mimic heat production [45]. A higher yield and stability of bioproducts can be achieved, such as in enzyme production, organic acids, bioactive compounds like antioxidants, vitamins, and other metabolites of interest, enabling the design of novel and functional foods with enhanced nutritional value [44]. SSF improves protein digestibility, enhances essential amino acid profiles, reduces anti-nutritional factors, generates desirable flavor compounds, and has been used in meat analog production [46].

The Chinese traditional SSF for traditional fermented foods has made significant progress, including raw material pretreatment, process parameter detection, mathematical model construction, and equipment innovation [47].

Some beneficial microorganisms in fermented foods, such as *Aspergillus* sp., *Rhizopus* sp., *Lactobacillus* sp., and *Bacillus* sp., grow very well in SSF [48]. Table 1 represents a comparative Analysis of SmF and SSF.

#### 3.1.3. PF

Precision fermentation utilizes genetically modified microorganisms to produce specific ingredients, including alternative proteins, enzymes, and flavors, for the food industry. The goal is to leverage Microbial cell factories to produce high-value functional food ingredients with high yields and purity while minimizing environmental impact [27]. PF is an alternative method for producing recombinant proteins, such as gelatin and collagen, for the food industry. Several technologies are employed, including CRISPR (Clustered Regularly Interspaced Short Palindromic Repeats), genetic engineering, and vector integration [49]. Animal hides, bones, and tendons are sources of collagen and gelatin proteins for the food industry. However, the processing of these biological materials, including cleaning, chemical treatments, and hydrolysis, can increase costs and slow down production. In this context, PF accelerates the process and improves production quality, while also reducing ecological impacts [50].

Milk proteins are of significant interest in infant nutrition, food manufacturing, and sports nutrition. Consequently, their production through PF has been rapidly expanding. Intensive research efforts have enabled several companies to achieve promising results in the production of bovine β-casein, for instance, in the development of cheeses entirely free from animal-derived ingredients. New Culture, a biotechnology company, has successfully produced mozzarella with texture, flavor, and melting properties comparable to those of its animal-derived counterpart [45].

Other examples of alternative proteins obtained through precision PF are egg white proteins (EWPs), such as ovalbumin, ovotransferrin, ovomucoid, ovoglobulin, ovomucin, and lysozyme. Some companies produce ovalbumin via PF using the fungus *Trichoderma reesei* and the yeast *Komagataella phaffii*. The same yeast is employed by the company EVERY to produce ovomucoid, a heat-stable protein applied in beverages. NOVONESIS is exploring the antimicrobial potential of lysozyme for use in food processing applications. Finally, sweet-tasting proteins derived from fungi, such as mycodulcein, are also being produced through PF to reduce sugar content in foods and beverages, thereby contributing to healthier products [51].

### 3.2. Natural Fermentation

Natural fermentation is a spontaneous biochemical process that transforms organic substances, such as carbohydrates, through the action of naturally occurring microorganisms, including bacteria and fungi. This process happens without the intentional addition of pure cultures or engineered strains. It is a traditional method used to produce a variety of fermented foods. While natural fermentation is culturally significant and often safe, it lacks standardization, which raises concerns for commercial scaling or export unless proper Hazard Analysis and Critical Control Points (HACCP) are implemented. In contrast to controlled fermentation, natural fermentation relies on the spontaneous activity of environmental or substrate-associated microbiota, making it more susceptible to microbial variability and potential contamination. While traditional foods like kimchi, Tempeh, or sourdough are generally considered safe due to their long-standing cultural use, they can occasionally harbor undesirable microorganisms or produce biogenic amines and mycotoxins if conditions are not optimal [52,53]. It is essential to point out that natural processes can be done through industry.

#### 3.2.1. Wild or Spontaneous Fermentation (WF)

This fermentation is based on natural processes that have been used for centuries, such as in the production of cheese, yogurt, bread, wine, and beer. Food fermentation can proceed spontaneously. It is driven by the autochthonous (native/indigenous) microflora of the raw food materials or processing environment, for instance, sauerkraut, kimchi, and certain fermented soy products [54,55].

Spontaneous food and beverage fermentations are characterized by the natural inoculation of microorganisms from raw materials, equipment, or the environment [52,53]. Most spontaneously fermented foods and artisanal beverages are produced through spontaneous generation, meaning there is no control over the microbiota or the substrate used. However, they are an essential source of bioactive compounds, including antioxidant compounds, bioactive peptides, short-chain fatty acids, amino acids, vitamins, and minerals [56]. In Asia, fermented foods and beverages play a significant role in cultural heritage [57].

#### 3.2.2. Traditional Fermentation (TF)

It is a natural process, but it is necessary to add starter cultures, known as culture-dependent ferments. Typical examples are summarized in Table 2. One method of performing a culture-dependent fermentation is backslopping, in which a small amount of a previously fermented batch is added to the raw food, similar to what is done in sourdough bread. The starters can be natural (back-slopping) or commercial [58].

#### 3.2.3. Symbiotic Fermentation (SF)

This fermentation involves multiple microorganisms working together in a mutually beneficial way. It can be present in several types of fermentation. Kombucha and kefir are examples. Each type of fermentation is selected based on the objective and desired characteristics of the final product. Fermented foods offer several health benefits, including immune system modulation, reduced serum cholesterol levels, antihypertensive effects, and anticancer and antidiabetic properties [59,60].

It is essential to clarify that traditional fermentation can be symbiotic, such as sourdough and kefir, or non-symbiotic (pure-culture fermentation, like some yogurts with a single strain). Kombucha fermentation involves a multi-species microbial community known as a symbiotic consortium of bacteria and yeast. It is produced by fermenting a sweetened tea infusion over 10–20 days. Besides the liquid fraction, Kombucha has a deposit, a biofilm synthesized during fermentation, consisting of reticulated cellulose and embedded microbial cells [61,62].

### 3.3. Fermentation Pathways and Industrial Applications in Food Biotechnology

Fermented foods are characteristic of each civilization. Several factors, including climate, local products, and agro-residues, influence this aspect. These features, along with the diversity of microbial metabolism, contribute to the production of a wide range of fermented foods. The advancement of biotechnology and industrial fermentation enhances industrial-scale production in the market [58].

The fermented Processed Food Market size was valued at USD 105.8 billion in 2023 [63]. Dairy, vegetables, grains, beverages, and meat products are examples of them. Fermentation in traditional food processing is a natural or controlled process. In industrial settings, it can be either natural or engineered using SmF or SSF, with or without engineered microorganisms, depending on the product and scale. Additionally, the global market for fermented ingredients, which can be utilized in the food industry, was valued at US$47.7 billion in 2024 and is projected to reach US$79.3 billion by 2030.

The ingredients include amino acids, organic acids, polymers, vitamins, and enzymes [59]. The market of food ingredients represents progress in fermentation technologies and manufacturing, enhancing the efficiency, quality, and versatility of fermented products. Modern techniques, including PF, bioengineering, and the utilization of microbial consortia, are enabling the scalable production of high-purity and stable bioproducts. Currently, with PF, it is possible to synthesize amino acids, enzymes, and vitamins with precise control over the process. This level of accuracy is especially valuable in the food, beverage, and animal nutrition sectors. In addition, new methodologies in encapsulation and stabilization techniques are protecting sensitive fermented products, such as enzymes and probiotics, extending and ensuring their viability during storage and consumption [31,60,64]. The integration of digital platforms and data analysis supports real-time monitoring and optimization of fermentation conditions, ensuring consistent and high-quality results [65].

Several metabolic pathways contribute to fermentation. Microalgae and fungi, including filamentous and unicellular yeasts, are classified as eukaryotes, while bacteria, including Archaea and the Bacteria domain, are prokaryotes. All of them have complex metabolic pathways, depending on the bioproduct formed. The most common fermentations are alcohol fermentation, lactic acid fermentation (homofermentative and heterofermentative), butyric acid fermentation, propionic acid fermentation, and acetic acid fermentation.

**Figure 3:**
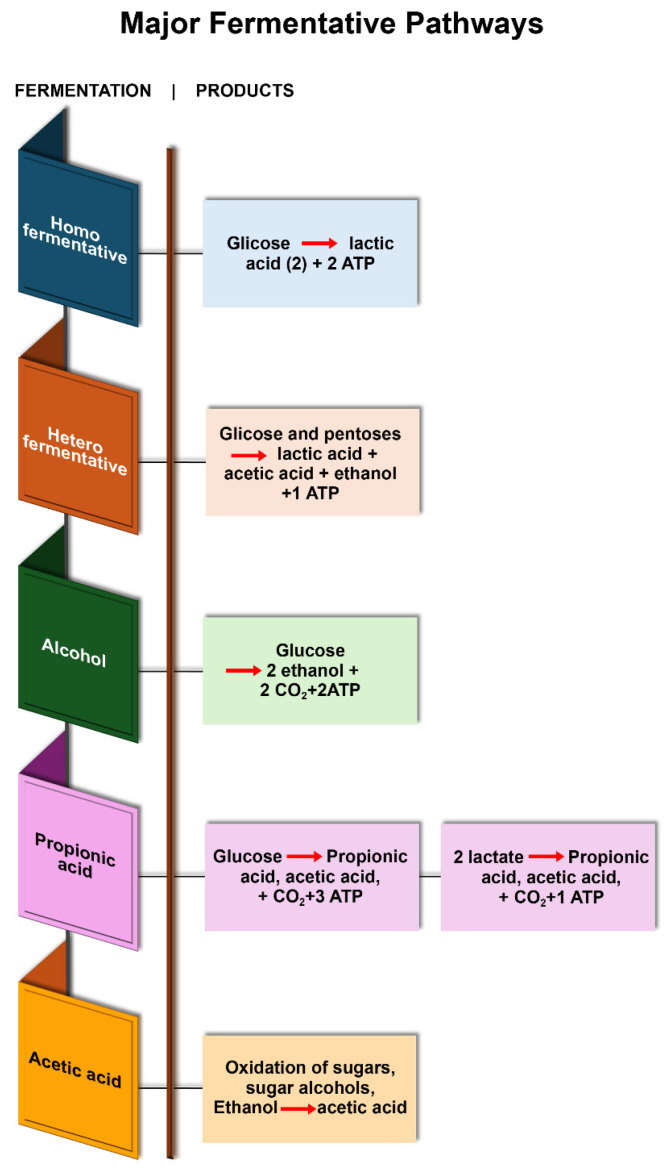
Major Microbial pathways used in food fermentation.

Fungal metabolites produce enzymes that are used in various food fermentation processes. Additionally, they can generate food ingredients such as amylase and galactosidase, as well as fatty acids, flavoring compounds, organic acids, pigments, and vitamins. Figure 3 summarizes the major microbial fermentation pathways involved in these processes. Both eukaryotic and prokaryotic microorganisms commonly utilize the Embden–Meyerhof–Parnas (EMP) pathway to break down glucose into pyruvate. Others, such as heterofermentative microbes, use the phosphoacetylase pathway to metabolize pentoses. Meanwhile, the Entner–Doudoroff pathway breaks down lactose molecules.

Homofermentation primarily produces lactic acid, which lends a mild flavor, and is utilized in yogurt, cheese, and pickles. Heterofermentation yields lactic acid, ethanol/CO_2_, and flavor compounds, which are beneficial for sauerkraut, kimchi, and sourdough production. Alcoholic fermentation is carried out mainly by yeasts (Saccharomyces cerevisiae), converting sugars into ethanol and CO_2_. In the food industry, it’s applied to produce beverages like beer, wine, and cider, as well as leavened products like bread, where CO_2_ creates dough expansion and ethanol evaporates during baking; Propionic fermentation is performed mainly by Propionibacterium species, which convert lactate into propionic acid, acetic acid, and CO_2_. In the food industry, it is most known for giving Swiss-type cheeses such as Emmental. The release of CO_2_ forms their nutty flavor and characteristic holes. Acetic acid fermentation is carried out by Acetobacter and related bacteria, which oxidize ethanol into acetic acid in the presence of oxygen. In the food industry, it is mainly used to produce vinegar (from wine, cider, or rice alcohol), contributing acidity, flavor, and preservation to foods, sauces, and condiments.

Pyruvate is a central hub for metabolic pathways, including glycolysis, the tricarboxylic acid (TCA) cycle, and the biosynthesis of amino and fatty acids. Pyruvate and its derivatives are essential for producing industrial compounds, including acetoin, 2,3-butanediol, butanol, butyrate, and L-alanine. Butyrate is often used as a feed additive and flavoring agent in animal nutrition [66,67].

Lactic acid bacteria (LAB) are widely explored as starter cultures in the food industry to enhance the gustatory, nutritional value, and flavor. Various LAB fermentation metabolites, such as lactic acid, aroma compounds, acetic acid, ethanol, bacteriocins, exopolysaccharides, and enzymes, are now widely explored in the food processing industry. The homofermentative process, in addition to the traditional final products summarized in Figure 2, can also produce acetaldehyde and hydrogen peroxide, which contribute to the aroma, flavor, and antibiotic effects in various food products.

Citrate metabolism plays a key role in *Lactococcus lactis* subsp. *lactis* (biovar diacetylactis) and *Leuconostoc mesenteroides* subsp. *cremoris* strains are widely used in the dairy industry. Citrate is cleaved by citrate lyase into oxaloacetate, which is decarboxylated to pyruvate by oxaloacetate decarboxylase. This metabolic pathway leads to the accumulation of intracellular pyruvate, which can be further converted to α-acetolactate and subsequently to diacetyl. Diacetyl is a major contributor to the buttery flavor and characteristic aroma of various fermented dairy products, including butter and certain cultured milk [68].

In Alcohol fermentation, another extensively explored fermentation process, pyruvate is reduced into ethanol via electron donation from NADH [69].

The major metabolic pathways represented in Figure 3 are present in fermented foods and in the production of ingredients and other metabolites for the food industry.

Below are some examples of fermented food and their fermentation pathways.

- Kombucha

Made with tea is an example of symbiosis between different microbial species, including bacteria and yeast, which allows three distinct fermentation processes to occur: alcoholic, lactic, and acetic. The process begins with sucrose, which is formed from fructose and glucose, catalyzed by an invertase from *Saccharomyces cerevisiae*. *Acetobacter* sp. and *Gluconobacter* sp. utilize these monosaccharides to produce organic acids, including acetic, gluconic, and glucuronic acids. The last improves liver function by promoting liver detoxification from xenobiotics [70]. The production of ethanol and acetic acid inhibits the growth of pathogenic bacteria. The polyphenol content present in black tea enhances the antioxidant effect. It promotes the production of bacterial cellulose, which forms a floating biofilm that allows bacteria and yeasts to coexist and oxygenate on its surface. This fermented drink can be considered a source of bioactive components, such as glucuronic acid, polyphenols, water-soluble vitamins (B1, B2, B6, B12, C), amino acids, minerals, and LAB [71].

Propionic acid, used as a food preservative, is also a naturally occurring compound found in some fermented foods. Bacteria, primarily the genus *Propionibacterium*, convert carbohydrates such as glucose, lactose, or ethanol into propionic acid. It is a major contributor to the characteristic nutty, sweet flavor of Swiss-type cheeses, including Emmental, Gruyère, and Appenzelle [72]. Butyric acid is a Volatile fatty acid, C4, that can be produced from agricultural residues as a high-value compound [73].

- Miso

Miso production utilizes SSF, which involves fermenting soybeans with *Aspergillus oryzae* (koji mold), lactic acid bacteria, and yeast in a low-moisture environment. It is an Anti-inflammatory food and supports cardiovascular health [74].

*A. oryzae* produces several enzymes, such as amylases, peptidases, and lipases. Specifically, amylase converts starch from rice into glucose. This fermentation process also facilitates subsequent lactic acid fermentation and alcoholic fermentation, which are carried out by lactic acid bacteria and yeast, contributing to the development of miso’s flavor [75,76].

A similar procedure is done with Tempeh. It is produced by SSF. The fungus *Rhizopus* spp. (mainly *R. oligosporus*) grows on cooked soybeans, forming a compact matrix with white mycelium. During fermentation, enzymes such as peptidases, lipases, and phytases break down proteins, lipids, and phytates, making Tempeh more nutritious and digestible [77].

It is essential to emphasize that the action of fungal enzymes breaking down macromolecules, such as proteins, polysaccharides, and lipids, is referred to as “fungal fermentation” in the food industry. It is an intense process involving the production of enzymes and aerobic respiration. There may be some secondary fermentation in areas with lower oxygenation.

- Natto

Produced by presoaked soybeans, which are cooked until tender, drained, cooled to 40 °C, and inoculated with a water suspension of *Bacillus natto*. The product is packed in a wooden box or polyethylene bag and incubated at 40–43 °C for 12–20 h. During the process, *B. natto* breaks down soybean macromolecules (proteins, lipids, and carbohydrates) through the secretion of enzymes such as peptidases, amylases, and lipases, generating more minor compounds. The bacterial enzyme-mediated hydrolysis of proteins into easily digestible peptides improves their nutritional value. In addition, the bacteria produce glutamic acid, which is responsible for the umami flavor, and polysaccharides that contribute to natto’s sticky texture [78,79]. Information about other fermented foods is briefly described in Table 2.

**Table 2:** Fermented food with fermentation pathway, bioactive compounds, and health benefits.

Fermented Food	Fermentation Pathway or Process	Fermentation Types Used	Main Bioactive Compounds	Health Benefits	Reference
Yogurt	Lactic fermentation	TF	Probiotics, peptides	Gut health, immune support	[80]
Sauerkraut	Lactic and homolacticfermentation	WF, NF (sometimes TF with starter)	Lactic acid, vitamins	Digestive aid, antioxidant properties	[81,82]
Kefir	Lactic and alcoholic fermentation	SF, TF or NF	Probiotics, peptides, and organic acids	Enhances gut microbiota, boosts immune function	[83]
Kimchi	Lactic fermentation	WF, NF, TF	Probiotics, vitamins, polyphenols	Supports gut health, anti-inflammatory properties	[74]
Tempeh	Fungal enzymes Lactic fermentation	SSF, TF	Isoflavones, peptides, prebiotics Vitamin B12	Improves digestion, supports bone health	[84]

TF—Traditional Fermentation; WF—Wild or Spontaneous Fermentation; NF—Natural Fermentation; SF—Symbiotic Fermentation; SSF—Solid-State Fermentation.

## 4. Food Additives and Ingredients

Food additives, according to the Codex Alimentarius, mean “any substance not normally consumed as a food by itself and not normally used as a typical ingredient of the food, whether or not it has nutritive value, the intentional addition of which to food for a technological (including organoleptic) purpose in the manufacture, processing, preparation, treatment, packing, packaging, transport or holding of such food results, or may be reasonably expected to result, (directly or indirectly) in it or its by-products becoming a component of or otherwise affecting the characteristics of such foods. The term does not include contaminants, substances added to food to maintain or improve nutritional qualities, or sodium chloride [85].

Novelty in food additives lies not only in replacing synthetic compounds with natural ones but also in the use of microbial and biotechnological processes that allow the development of more environmentally friendly and health-promoting alternatives. Food additives are a widely used strategy to preserve and enhance the quality of industrial food. It encompasses various categories, including coloring agents, preservatives, emulsifiers, thickeners, antioxidants, carriers, acids, acidity regulators, anticaking agents, antifoaming agents, bulking agents, emulsifying salts, firming agents, flavor enhancers, foaming agents, gelling agents, glazing agents, humectants, and sweeteners [86].

For a long time, chemical additives produced by chemical processes have been the most scalable and cost-effective. Nevertheless, microbial additives have been increasingly recognized as a potential substitute for chemical additives, particularly due to their sustainability. They also provide a healthier alternative to food additives. However, the need for stricter regulation to prevent the dangers associated with the improper use of certain microorganisms that are harmful to health increases.

### 4.1. Chemical Additives

Customers demand food with non-toxic and healthy additives, as several of these additives are reported to harm human health or well-being [87]. In this context, some chemical additives are reported to cause human dysfunction; Table 3 summarizes some of them. However, a study highlights that it is essential to note that although adverse effects are attributed to certain food additives, more comprehensive research based on clinical trials is necessary [88].

### 4.2. Microbial Bioproducts as Food Additives

There are increasing opportunities for the development of new, more sustainable, and environmentally friendly additives. In this context, microbiology provides numerous bioproducts that can be harnessed for this purpose. Microbial enzymes, organic acids, texturizers, stabilizers, food colorants, sweeteners, flavorings, aromas, and functional and nutritional bioproducts are examples of microbial products used as food additives and will be discussed in this section.

Regarding the use of microbial additives in food and beverages, it is essential to note that safety guidelines established by official regulatory agencies must be strictly adhered to and thoroughly investigated. This is because some proteins, including enzymes used as food additives, can interact with the human immune system, leading to sensitization and allergic reactions [102]. In general, common food allergens include gluten, peanuts and tree nuts, cow’s milk, eggs, among others [103]. However, microbial products added to foods may also represent new potential sources of risk.

To ensure microbial safety and functional reliability, the selection of production strains involves a combination of phenotypic screening and molecular profiling. Safety evaluations typically include tests for toxigenicity, the presence of antimicrobial resistance genes, biogenic amine production, and hemolytic activity. Genomic sequencing plays a key role in confirming strain identity, genetic stability, and the absence of virulence factors. At the same time, metabolic profiling is used to assess enzyme yield, byproduct formation, and the regulation of metabolic pathways under industrial conditions [104]. These strains are generally recognized as safe by international regulatory agencies such as the European Food Safety Authority (EFSA) and the U.S. Food and Drug Administration (FDA). They are included in lists such as GRAS (Generally Recognized as Safe) or QPS (Qualified Presumption of Safety), which reinforces their suitability for safe and practical applications in the food industry.

In Brazil, regulatory bodies such as the National Health Surveillance Agency (ANVISA) have established official lists of microbial strains approved for use in the production of food additives. These strains must be genetically stable, non-pathogenic, and non-toxigenic, ensuring consumer safety and consistent performance in large-scale industrial processes [105]. A brief comparison between important international regulatory frameworks regarding food additives is shown in Table 4.

#### 4.2.1. Microbial Enzymes

Microbial enzymes are considered biotechnological tools applied in the food industry. More than 3000 microbial enzymes have been identified through genomic and proteomic analyses, highlighting their vast biochemical diversity and industrial potential [117]. The advantages of these biocatalysts are numerous, including high catalytic efficiency, stringent substrate specificity, and compatibility with environmentally sustainable processes, which enable them to replace energy-intensive chemical methods while reducing the generation of hazardous byproducts [118,119]. Their biodegradability and ability to function under mild physicochemical conditions (pH and temperature) further reinforce their role in sustainable food production chains.

Basketter and Kimber (2022) [120] discussed how enzymes may contribute to the development of immune system overreactions mediated by IgE antibodies, leading to allergies through sensitization of the skin and respiratory tract. For instance, scientific evidence has demonstrated that microbial transglutaminase, used as a food additive to modify functional properties, may be a potential trigger of autoimmune diseases [121]. Although cases of allergenicity caused by food enzymes have been reported, assessments also demonstrate the safety of these bioproducts. A recent study revealed that processes involving α-amylase from a genetically modified *Bacillus licheniformis* strain do not pose additional safety concerns, as the enzyme is considered safe under the intended conditions [122].

Among biological sources, microorganisms such as filamentous fungi and bacteria are particularly valuable due to their rapid growth, cost-effective cultivation, and amenability to genetic manipulation [31,123]. Notwithstanding their recognized biotechnological value, the integration of microbial enzymes into food applications is contingent upon stringent safety evaluations. Although many production strains are classified as GRAS or QPS, international regulatory guidelines emphasize the need for thorough assessments that address concerns related to allergenicity, immunotoxicity, toxicology, antimicrobial resistance, and metabolic stability. International agencies as EFSA and FDA, as well as national authorities such as ANVISA, have established clear guidelines requiring genetic stability, non-pathogenicity, and non-toxicity of production strains to guarantee consumer safety and functional reliability [104,124].

In this technical and regulatory context, microbial enzymes have been consolidated in the food sector, reflecting both their technological relevance and their longstanding record of safe use.

To improve readability and synthesis, the most representative and additional enzymes are summarized in Table 5, which highlights their microbial sources, primary biochemical functions, and industrial applications.

#### 4.2.2. Microbial Organic Acid

Industries have chosen organic acids from microorganisms because they present better cost feasibility and are more environmentally friendly when compared to chemical production [177]. Additionally, the development of technology focused on production enhancement in microbiology, including bioprocesses and molecular tools, has led to a growing trend in the use of these microbial acids in the food industry. Among the most relevant microbial organic acids are acetic, lactic, propionic, succinic, and citric acids. Their main functions in food systems, along with the principal microbial producers, are summarized in Microbial organic acids, particularly acetic, lactic, propionic, succinic, and citric acids, play key roles in food preservation, flavor enhancement, and technological functionality. Their principal functions in food systems, along with the representative microbial producers, are summarized in Table 6.

#### 4.2.3. Other Microbial Bioproducts

- Texturizers and stabilizers

Microorganisms serve as valuable sources of polysaccharides, emulsifiers, and surfactants for food additives that function as texturizers and stabilizers. Organizing these additives based on their microbial origin provides a clearer understanding of their sources and applications.

- Food colorants

Microbial pigments are another trend in food additives, as many synthetic colorings are associated with harmful properties, such as carcinogenic and allergenic effects. It is essential to highlight that these pigments possess many other properties, including antioxidant activities, which are superior to those of traditional synthetic food additives, such as butylated hydroxyanisole (BHA) and butylated hydroxytoluene (BHT). Additionally, these pigments have been shown to exhibit anticancer, immunosuppressive, anti-inflammatory, and antiproliferative properties [202].

- Sweeteners

Advances in microbial biotechnology and metabolic engineering have expanded the range of natural sweeteners suitable for food applications. Natural sweeteners are increasing in the market because they are more sustainable and healthier. Plants and microorganisms are sources of natural sweeteners, although microorganisms are economically more viable [203].

- Flavoring and aroma

Microorganisms play a fundamental role in the development of flavors and aromas in foods, mainly through fermentation and biocatalysis processes. They can synthesize a wide range of volatile and non-volatile compounds, including organic acids, alcohols, aldehydes, ketones, esters, and amino acids, that contribute to the sensory quality of foods [204].

Furthermore, traditional fermentation and microbial inoculants have been applied to improve the flavor profile of agricultural fruits [205], while microbial conversion of agro-industrial waste has been employed as a sustainable strategy for producing natural flavoring agents [206].

- Functional and nutritional bioproducts

Beyond sensory improvements, microalgae and algae provide nutritional and health-promoting benefits, supplying essential metabolites such as vitamins, minerals, fatty acids, and prebiotic compounds. Its incorporation into foods has led to an increase in nutritional value and consumer acceptance. However, aspects related to safety, sensory acceptance, and regulation still represent challenges for wider industrial adoption [207].

Together, microbial bioproducts encompass a broad range of applications, including stabilizers, pigments, sweeteners, flavoring agents, and functional ingredients. Their main functions, microbial sources, and industrial applications are summarized in Table 7.

### 4.3. Probiotics, Prebiotics, and Postbiotics

Ingredients such as probiotics, prebiotics, and postbiotics serve as food additives or supplements depending on the legislation of each country. For example, in the United States, the Food and Drug Administration (FDA) considers prebiotics to be a food additive or Generally Recognized as Safe (GRAS) [219]. In Brazil, ANVISA regulates the use of probiotics in foods and dietary supplements, while prebiotics are currently regulated as bioactive substances within the framework of dietary supplements [220]. It is essential to note that prebiotics and postbiotics are relatively recent concepts, and standardized definitions for these terms have yet to be established. Most of this problem is attributed to the development of new substances and advancements in knowledge of the gut microbiome. However, nowadays, the World Health Organization/Food and Agriculture Organization of the United Nations (WHO/FAO), the International Scientific Association of Probiotics and Prebiotics (ISAPP), and the Global Prebiotic Association (GPA) are active in establishing these terms [221].

#### 4.3.1. Probiotics

According to the WHO and FAO, probiotics are “live microorganisms that, when administered in adequate amounts, confer a health benefit on the host”. This definition is accepted by the ISAPP and other scientific institutions worldwide [222].

Lactic acid bacteria primarily represent probiotics, such as *Lactobacillus*, *Leuconostoc*, *Weissella*, *Lactococcus*, *Enterococcus*, *Streptococcus*, and *Pediococcus*; however, other genera, such as *Bacillus*, and some yeasts, like *Saccharomyces boulardii*, *S. cerevisiae*, and *Candida* spp., can also qualify as probiotics [223,224]. These microorganisms are recognised for their health-promoting benefits. They are naturally present in fermented food such as sourdough, buttermilk, kombucha, miso, kefir [224], and are commonly used in food products or the biomolecules produced during fermentation. With the growing market demand for probiotics, there is an increasing need to establish the criteria that qualify microorganisms for this status. It is essential to demonstrate the absence of toxicity, virulence, and resistance to low pH levels, gastric enzymes, and bile salts.

Additionally, probiotic adhesion capacity and approval from clinical trials are key factors in determining the success of a product. To ensure consumer safety, the WHO and the FAO emphasize that the evaluation of probiotics should rely on molecular information from specific strains, as the beneficial properties of probiotics are closely linked to their genetic identification [223]. For instance, the genus *Bacillus* comprises a group of Gram-positive bacteria that encompasses a wide range of strains, including some that are pathogenic and others that are probiotic. Certain strains within the *B. cereus* group, including *B. cereus*, *B. anthracis*, *B. thuringiensis*, and *B. mycoides*, produce toxins; therefore, characterizing these strains at a detailed level is essential. Furthermore, research has highlighted the importance of *Bacillus* in various fermented foods, such as bread, beverages, and traditional dishes. This highlights the need for a more comprehensive understanding of the genetic variability within this genus [148].

Lactic acid bacteria are the most studied class of probiotics, as they are the microbiota well-adapted to cereal fermentation, such as sourdough and beverages [225,226]. During these fermentations, various biomolecules with bioactive properties are produced, including peptides, enzymes, organic acids, exopolysaccharides, and vitamins. Moreover, these compounds are related to multiple technological aspects in food, including flavoring, aroma, preservatives, and emulsifiers, as well as potential health or well-being benefits for consumers. Cancer prevention, stabilization of cholesterol levels, anti-inflammatory effects, and the reduction of allergenic substances such as proteins and fermentable oligo-, di-, and monosaccharides and polyols (FODMAs) are among the beneficial effects that enhance the nutritional value of foods [223,224].

Specific industrial food processes can compromise the viability of probiotics, including exposure to extreme temperatures, pH fluctuations, or inadequate storage conditions. Moreover, the digestive process in the stomach’s acidic environment, combined with the presence of bile salts, can be a challenge to overcome. Technologies such as encapsulation using biopolymer hydrogels and 3D printing for encapsulation are examples of promising technologies that can help overcome these barriers to achieve functional foods with probiotic [227,228].

Nevertheless, studies have highlighted the dangers of consuming probiotics without supervision, such as *Lactobacillus* sp., *Bifidobacterium* sp., the probiotic strain of *Bacillus*, and *S. cerevisiae* var. *boulardii*, which can appear pathogenic in certain situations. For instance, endocarditis, sepsis, bacteremia, liver abscess, pneumonia, intra-abdominal abscess, and cholecystitis were associated with *Lactobacillus* sp., *L. rhamnosus*, *L. rhamnosus* GG, and *L. acidophilus*. Some cases of bacteremia and sepsis were also associated with *Bifidobacterium* sp., *B. longum*, *B. breve*, *B. clausii*, and *B. subtilis*. Additionally, some cases of fungemia by *S. cerevisiae* were observed [229].

Recent research points to a liver abscess caused by *L. plantarum* in a 62-year-old female patient with pancreatic cancer. The treatment for pancreatic cancer utilizes the method of endoscopic retrograde cholangiopancreatography (ERCP), which is favorable for this type of infection due to the disruption of the hepatobiliary system and duodenum [230]. It is important to note that all cases related in the literature are linked to people with immunosuppression, AIDS, prolonged hospitalization, surgical interventions, transplantation, and use of ERCP. Concisely, serious illnesses [229,231,232].

#### 4.3.2. Prebiotics and Dietary Carbohydrates

One of the widely accepted definitions of prebiotics is “a substrate that is selectively utilized by host microorganisms conferring a health benefit.” This broad concept enables innovation by expanding the classification beyond carbohydrates to include non-carbohydrate compounds such as polyphenols and polyunsaturated fatty acids (PUFAs). Moreover, the definition applies to multiple hosts, including humans and animals, and extends beyond the gut microbiota to encompass microbial ecosystems of the skin, oral cavity, and urogenital tract [221].

To understand the role of prebiotics more clearly, it is essential to distinguish them from dietary carbohydrates. At the same time, all prebiotics are dietary carbohydrates or related substrates; not all nutritional carbohydrates function as prebiotics. Dietary carbohydrates encompass a wide range of molecules, including digestible sugars like glucose and starch, as well as non-digestible fibers like cellulose and pectin. However, only a subset of non-digestible carbohydrates—specifically those that reach the colon intact and are selectively fermented by beneficial gut microbes—qualify as prebiotics [221].

For instance, cellulose is a non-digestible carbohydrate that provides bulk and aids in bowel movement, but it is not fermentable and does not selectively stimulate beneficial microbes. Thus, it is not classified as a prebiotic. In contrast, inulin, fructooligosaccharides (FOS), and galactooligosaccharides (GOS) are selectively fermented by *Bifidobacterium* sp. and *Lactobacillus* sp. species, resulting in the production of health-promoting short-chain fatty acids (SCFAs), such as butyrate, propionate, and acetate. These compounds help maintain gut barrier integrity, modulate immune responses, and serve as energy sources for colonocytes [233,234].

A wide range of compounds are now recognized as prebiotics, including conjugated linoleic acid (CLA), PUFA, FOS, inulin, GOS, MOS, XOS, human milk oligosaccharides (HMOs), phenolics, phytochemicals, fermentable dietary fibers, resistant starch, acacia gum, yeast-based substrates, botanicals, amino acids, omega-3 fatty acids, polyphenols, and resistant dextrin [219,221,235].

Microorganisms play a crucial role in the synthesis of these bioactive prebiotics. Bacterial exopolysaccharides (EPS), SCFAs, various oligosaccharides, and microalgae-derived compounds significantly contribute to gut microbiota modulation [225,226]. Specific strains of *Lactobacillus* sp. and *Bifidobacterium* sp. are known to produce EPS that promotes the growth of other beneficial bacteria [236]. For example, *Leuconostoc mesenteroides* synthesizes dextran, which enhances the proliferation of *Bifidobacterium* [237,238], while *Lactobacillus reuteri* produces levan, a fructan that supports *Lactobacillus* and inhibits the growth of pathogens [239].

*Bifidobacteria* play a crucial role in gut carbohydrate metabolism, particularly in converting lactose into GOS, which selectively promotes the growth of beneficial microbial populations. A recent study observed *B. bifidum* with high performance in producing β-galactosidase, an enzyme involved in GOS production. This β-galactosidase exhibits properties suitable for use in dairy products, yielding a high GOS content with a lower allergenicity risk compared to commercial GOS, due to its linkage preference [240]. Species such as *B. longum* subsp. *infantis* and *B. bifidum* possess genes encoding β-galactosidase and related enzymes that enable this bioconversion [241]. In contrast, others, such as *B. adolescentis*, show limited GOS production due to differences in glycoside hydrolase gene content [242]. Recent in vivo studies in murine models have shown that GOS supplementation significantly increases *Bifidobacterium* abundance, improves intestinal tolerance to lactose, and enhances gut barrier function, effects mediated by modulation of the microbiota rather than increased lactase activity [243].

Beyond *Bifidobacterium* sp., other microbial strains contribute to prebiotic diversity. Yeasts, such as *Aureobasidium pullulans*, produce FOS through fermentation, which supports the growth of *Lactobacillus* species [154,244]. Fungi, such as *Saccharomyces cerevisiae* and medicinal mushrooms, synthesize β-glucans that modulate both gut microbiota and immune responses. Additionally, MOS derived from yeast cell walls helps reduce pathogen colonization and promote the growth of probiotics [245]. Together, these findings highlight the diverse metabolic strategies employed by microbial taxa in producing bioactive oligosaccharides relevant to host health.

Microalgae, such as *Chlorella* and *Spirulina*, are rich in sulfated polysaccharides with proven prebiotic effects, promoting the growth of beneficial bacteria and suppressing pathogens [246,247]. Their polyunsaturated fatty acids and antioxidants also reduce intestinal inflammation and oxidative stress.

Furthermore, microorganisms can biosynthesize lipids such as oleic acid, GLA, ARA, EPA, and DHA, offering sustainable alternatives to fish or plant-based sources. For example, *Mortierella alpina* 1S-4 is commercially used to produce ARA-rich oil for infant formulas, and products such as New Harvest and SUNTGA40S exemplify microbial lipid innovations [237].

Recent studies have shown that *Lactobacillus plantarum* 13-3 and 2-3 can biotransform linoleic acid into conjugated linoleic acid (CLA), fatty acid methyl esters (FAME), and oxygenated long-chain fatty acids, which exhibit antimicrobial and anti-inflammatory properties [248,249]. *Saccharomyces cerevisiae* var. *boulardii* CNCM I-745 produces trehalose, glucuronic acid, GABA, and phenolics in the presence of inulin, enhancing immune modulation in synbiotic matrices like ice cream [238]. *Bacillus* spp. also generate antioxidant and anti-inflammatory metabolites, including 2-propanone and phthalic acid derivatives [250].

More recently, exogenous hydrolases, such as peptidases and lipases, have been investigated for their potential as prebiotics due to their observed ability to enhance animal gut microbiota composition [251].

Some advancements have been observed in producing functional foods that combine probiotics with prebiotics using co-encapsulation techniques. For instance, a typical Iranian drink, Doogh, was made with co-encapsulated *Lactobacillus plantarum* LS5 with inulin. Other examples included soymilk with co-encapsulated *Lactobacillus rhamnosus* GG with resistant and waxy starches. Cupcake co-encapsulated *Lactobacillus plantarum* ATCC8014 with maltodextrin. All of them increased the viability of probiotics in the final food [252].

Despite significant progress, several challenges persist. Strain-specific variability in metabolite production, lack of clinical validation in humans, and regulatory uncertainty regarding novel prebiotics still limit broader application. Advanced omics approaches and precision formulation strategies are needed to optimize the health benefits and delivery of next-generation prebiotics.

#### 4.3.3. Postbiotics

Postbiotics are structures derived from microorganisms after cellular death, including all metabolites and cellular components that might promote health benefits to the host. For instance, lactocepin, teichoic acids, GOS, SCFAs, acetate, butyrate, dimethyl acetyl-derived plasmalogen, lactate, propionate, propionic acid, lactic acid, citric acid, acetic acid, B-complex vitamins, peptides, amino acids, and peptidoglycan [253]. It is essential to note that metabolites are considered postbiotics only when they are associated with biomass. Moreover, postbiotics can utilize any microorganisms, even pathogenic ones, since they are inactivated during the safety process [254,255].

The increasing interest in food science regarding postbiotics is due to the possibility of obtaining food improvements without the inconvenience of using live microorganisms, such as probiotics, which require storage, maintenance during food industrial processes, and preservation after the food process. Moreover, it is essential to note that some technologies for obtaining postbiotics must guarantee the postbiotic functional structure [255].

Japan has been a leader in the use of postbiotics for over a century. Many Japanese food products, such as juices, ice creams, natto, miso soup, and supplements, include inactivated forms of lactic acid bacteria or bifidobacteria. Although most of these products do not explicitly claim health benefits, a few—specifically two fermented milk drinks and a tablet—have received approval under Japan’s Foods with Function Claims (FFC) system. This system requires scientific evidence to support any functional health claims [255].

Research suggests that functional foods containing postbiotics may help with obesity through various mechanisms, including increased energy expenditure, decreased fat cell formation and differentiation, reduced food intake, inhibition of lipid absorption, regulation of lipid metabolism, and correction of gut dysbiosis [256]. Additionally, other studies have demonstrated the benefits of postbiotics in managing obesity, diabetes, lipid dysfunction, postprandial blood glucose levels, constipation, strengthening the immune system, promoting oral health, and supporting skin health [255]. Notably, lysate from *Bacillus velezensis* Kh2-2, derived from Korean food, has shown immune-enhancing effects [257]. Furthermore, ongoing research on postbiotics produced by *Lactobacillus acidophilus* has demonstrated that lactic acid, in specific concentrations, exhibits antimicrobial effects. However, further studies are needed to determine their suitability as food additives [258]. For example, yogurt supplemented with postbiotics demonstrated antimicrobial action against *Listeria monocytogenes* and *E. coli* after 21 days of refrigeration [259]. Moreover, a study evaluated the use of postbiotics from probiotics for food preservation. It concluded that they were efficient in inhibiting pathogenic bacteria, while also highlighting the importance of validating their efficacy in real food systems [260].

The new generation of functional food previews the synergistic use of postbiotics with probiotics and prebiotics. For instance, prebiotics such as FOS can selectively stimulate probiotics, increasing the yield of postbiotics, including SCFAs and bacteriocins, which in turn improve gut health [259].

Several challenges are associated with using these additives in food. Many studies supporting their human benefits are conducted in vitro or on animals rather than through clinical trials. Additionally, advancements in technology are needed to enhance industrial processes, such as characterizing new microorganisms and their postbiotic molecules, as well as developing effective methods for their extraction [255].

Nevertheless, a study concluded that postbiotics obtained from functional foodsincluding short-chain fatty acids, exopolysaccharides, enzymes, cell wall components, and other microbial metabolitesexert diverse health-promoting functions such as anti-inflammatory, immunomodulatory, antioxidant, and anticancer effects. Together, these activities support intestinal barrier integrity, regulate immune homeostasis, and limit colorectal cancer progression by inducing apoptosis, modulating oncogenic signaling, and exerting epigenetic control. Although preclinical studies highlight the potential benefits of postbiotics for gut health, their clinical application still requires standardized definitions and protocols, thorough safety evaluations, clear regulatory frameworks, and solid clinical validation. In addition, interdisciplinary collaboration, together with multi-platform approaches and advanced multi-omic technologies, will be crucial to developing effective, safe, and sustainable adjuncts for colorectal cancer prevention and therapy [261].

While microbial additives offer advantages such as biodegradability, scalability, and reduced environmental impact, their effective use in the food industry depends on multiple technological factors. Compared to chemical synthesis, microbial production often requires less energy and generates fewer toxic by-products. However, challenges remain, including strain optimization, substrate selection, and yield efficiency, particularly for large-scale fermentation processes. Advances in biotechnology, particularly in molecular tools and metabolic engineering, have significantly enhanced microbial productivity, as exemplified by the case of lactic acid, which is now predominantly produced through fermentation. Microbial enzymes and biocatalysts have also expanded applications in food restructuring and flavor generation, although safety concerns and regulatory scrutiny persist due to potential allergenicity or altered bioavailability. Thus, the growing preference for microbial sources is not only a matter of sustainability but also of technological feasibility, made possible by recent bioprocess innovations discussed earlier in this article.

## 5. Food Preservation

Food preservation is a crucial topic in the food and beverage industry, as it controls microbial growth to prevent microbial spoilage, minimize economic loss, and ensure food safety for consumers. There are different ways to prevent microorganisms from contaminating food products, including physical, chemical, and biological approaches [262]. In recent years, artificial intelligence has also proven to be a powerful tool in food preservation [263].

Salts and organic acids, such as sodium chloride and sorbic acid, are common food preservatives in the industry. These additives prevent microbial spoilage by disrupting the balance of protons and anions, which affects cell membranes, enzymes, RNA, and DNA, and consequently inhibits crucial cell functions. Recent research has identified the potential for producing the food additive sodium erythorbate, which serves as both a preservative and an antioxidant. This additive is produced using *Pseudomonas plecoglossicida*, which is cultivated on rice contaminated with cadmium [264].

However, tolerance to these compounds has been reported in the literature for some food pathogens after continuous exposure [265]. Additionally, studies have reported that, in addition to tolerance to these chemical preservatives, some toxicity levels and risks to human health may be associated with prolonged consumption of these agents [265]. Due to this problem, incessant studies aim to develop new preservatives for the food industry, preferably with natural-based agents, to meet consumer preferences for healthier food and products.

### 5.1. Plant-Derived Preservatives

Plant-derived bio compounds with antimicrobial properties can be used as alternatives in applications as preservatives. These agents, which can be found in essential oils, polyphenols, and thiols, for example, have already been studied in the literature due to their bioactive properties, such as anti-tumoral, anti-inflammatory, and antioxidant actions, as well as potential antimicrobial properties. They are then being investigated as promising preservatives for use in food products. These biomolecules control microbial growth by disrupting the cell membranes of microorganisms, inhibiting cell division, enzymes, and other mechanisms [248,266]. Additionally, plants can also produce antimicrobial peptides, such as defensins and thionins, which have been investigated in the literature as potential food preservatives against common microbial pathogens found in food products [267]. Antimicrobial peptides will be further described in the following subsections.

It is crucial to note, however, that these compounds face several challenges for large-scale application due to their sensitivity to environmental conditions, high costs, and time-consuming extraction techniques, as well as the use of organic solvents in the process for obtaining low quantities of bioactive compounds [266].

### 5.2. Microbial Preservatives

#### 5.2.1. LAB Microorganisms

**Figure 4:**
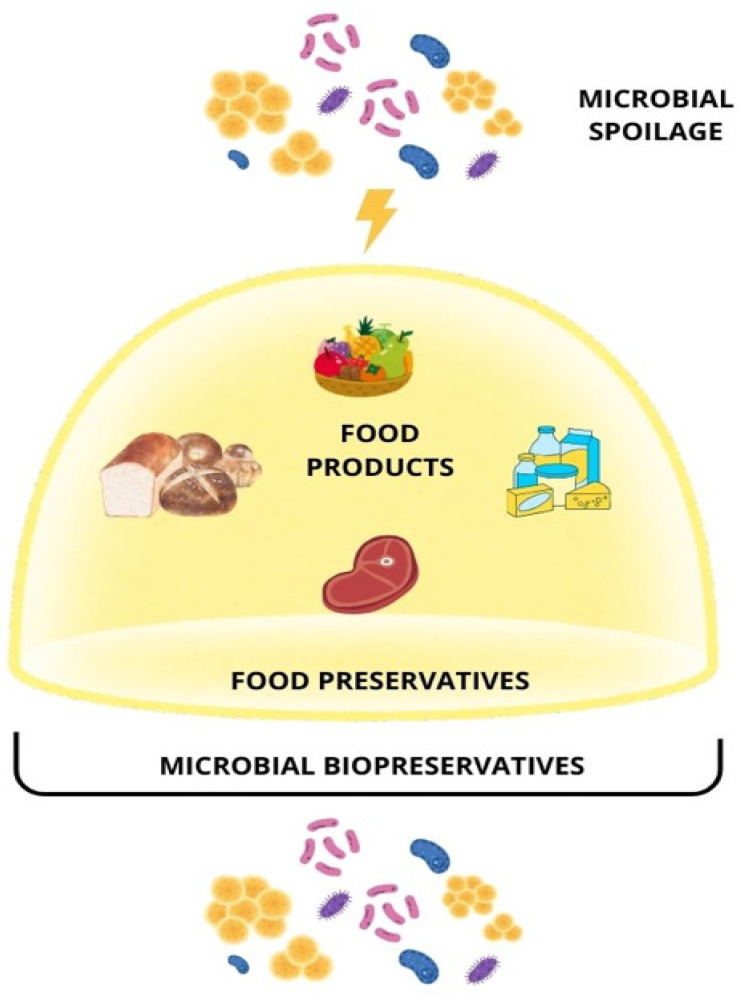
Representation of microbial bio-preservatives in food products acting as a barrier against microbial spoilage.

In this scenario, microbial agents play a crucial role in food preservation technology, being one of the leading causes of food spoilage (Figure 4). For example, LAB microorganisms comprise a group of Gram-positive, anaerobic, or facultatively anaerobic bacteria primarily known for producing lactic acid through fermentation and playing a significant role in food fermentation [249]. These bacteria are widely known for their beneficial effects in food products. In the baking industry, for example, lactic acid bacteria and specific yeasts act at different stages of the baking process, from preparing the bread dough (as inoculum and starters) to the preservation process and spoilage control [262], due to the secretion of volatile organic compounds, such as these. It was demonstrated that *Limosilactobacillus fermentum* IAL4541 and *Wickerhamomyces anomalus* IAL4533 exhibited potential bio-preservative properties in panettone products through a proto-cooperation mechanism [268]. These strains exhibited great preservative activity due to the production of bacteriocins and organic acids (lactic, acetic, formic, caproic, butyric, and propionic) during fermentation, consequently lowering the pH of the sourdough environment. The benefits of organic acids and low-molecular-weight metabolites produced by lactic acid bacteria have also been reported for other segments in the same industry, such as aquatic food [269] and dairy products [270].

#### 5.2.2. Antimicrobial Peptides and Other Metabolites

Besides their importance as food additives, LAB microorganisms have also been studied because they produce antimicrobial compounds, such as peptides, which can benefit food and beverage products [271,272]. Antimicrobial peptides are compounds found in animals, plants, and even microorganisms that protect against microbial pathogens, usually by acting as effective membrane disruptors of bacteria, yeasts, and filamentous fungi [273].

Regarding this subject, some microorganisms produce antimicrobial peptides, such as nisin (from *Lactococcus lactis*) and natamycin (from *Streptomyces natalensis* and other species) [274,275], as examples. These two molecules exhibit effective action, even at low concentrations, against bacterial and fungal agents and are used as preservatives in various food products, including meat, dairy products, fruits, and vegetables [274]. Thus, research investigating antimicrobial peptides with potential applications in the food industry has been trending in scientific literature, reporting new microbial agents, such as *B. subtilis* [276], *B. velezensis* [277], and *Enterococcus faecalis* [278]. Moreover, bacteriocins from LAB microorganisms are considered prospective, safe, and sustainable food additives in many countries, including China, Japan, India, Korea, Brazil, Russia, Turkey, Africa, Spain, Indonesia, and Vietnam, where some lactic acid bacteria or their bacteriocins are permitted for use in industrial food production. For instance, nisin is a bacteriocin widely used in meat, dairy products, coffee, tea, soy sauce, canned goods, and vegetable protein-based products [272]. These antimicrobial molecules also appear as an essential tool for sustainable control of post-harvested fruits [279]. Riolo and colleagues [280] reported novel bioformulations of antifungal compounds (mainly acids) obtained by two lemon peel isolates of *Lactiplantibacillus plantarum* (N3B2 and M2B2), which showed broad inhibitory activity against postharvest pathogens. Similarly, metabolites present in concentrated cell-free supernatants from the LAB strain CFS24 were able to reduce by 80% the incidence of blue molds (*P. digitatum* and *P. italicum*) in postharvest control of lemon fruits [281]. In another study, a yeast agent, *Candida oleophila*, was able to control green mold infection caused by *P. digitatum* under various conditions [282]. These findings highlight the promising potential of antimicrobial compounds as effective and sustainable alternatives for managing postharvest fruit biodeterioration, thereby extending the shelf life of these products. An interesting point to note is that studies on antimicrobial peptides have also aligned with future food market trends, such as the growing consumption of cultured meat (also known as cell-based or lab-based meat). As reported by Yakir and colleagues [283], the use of random antimicrobial peptide mixtures can present a consistent barrier against food pathogens, such as *L*. *monocytogenes*, without showing any cytotoxicity. These results suggest that antimicrobial peptides could be alternative agents to antibiotics and also highlight the promising application of these biomolecules in a future chain from the food market.

#### 5.2.3. Microbial Biopolymers

Microbial biopolymers are another source of biopreservation technology that the food industry has investigated [284]. Pullulan is a microbial biopolymer with maltotriose as repeating units in α-1,4 and α-1,6 glycosidic-linkage pattern, produced by the fungus *Aureobasidium pullulans* [285,286]. The food industry has already used pullulan in various food formulations. However, it is also a promising agent for preventing food spoilage due to its sensory aspects [286]. Wani et al. (2024) [287] reported that 3% pullulan edible coatings exhibited satisfactory preservative properties, extending the shelf life and maintaining the quality of post-harvest cherries for up to 20 days of storage. Besides fruits, pullulan has been investigated in composites and formulations for the preservation of other food products, such as rice [288], bread [289], and meat [290,291].

Xanthan gum is another microbial biopolymer (*Xanthomonas campestris*) that is already used as an ingredient in the food industry and has recently been studied as a food preservative, yielding promising results for various food products [292,293]. Xanthan gum also appears as a component of packaging biofilm in the food industry due to its properties, such as mechanical properties, barrier properties, antimicrobial properties, and biodegradability, as well as its adaptability as an intelligent packaging when incorporated with other bio compounds to extend the shelf-life of food products more efficiently [294]. As an example of this topic, a work demonstrates that xanthan gum and pectin-based films presented enhanced physicochemical properties, such as improved water resistance and tensile strength, after the addition of grapefruit essential oil to the formulation [295].

#### 5.2.4. Phages and Other Microbial Technologies

Microbial technology, utilizing bacteriophages (or phages), can also help prevent foodborne illnesses caused by common pathogens by infecting and disrupting bacterial cells. Phages have been sought in scientific literature as an innovative methodology and sustainable biocontrol agents in the food industry due to their laboratory-based manufacturing and specific target mode of action, without compromising side factors in the food production chain. Mohammadi and colleagues [296] critically reviewed the characteristics of the upstream and downstream processes required for the large-scale production of phages while also highlighting some gaps in the regulations governing the use of this technology that need improvement to advance its application in industry.

*P. aeruginosa*, which poses a significant risk in the food industry, for example, was the target of a recent study that identified a bacteriophage named Phage vB PaeM HUST 1, which is effective as a food additive to combat this bacterium in milk and beef [297]. Qin and colleagues [298] recently investigated the potential role of phage vB_Sal_M467 targeting *Salmonella enterica serovar* Manhattan, another critical food pathogen. The studied phage exhibited excellent results in inhibiting the growth of bacterial pathogens in lettuce, milk, and apple juice at various temperatures, suggesting its potential as a novel antibacterial agent for food preservation. *Listeria*-specific bacteriophages have also been studied as antimicrobial agents for food products, such as cheese and meat products, where *Listeria monocytogenes* often contaminates after pasteurization processes, posing a risk to human health [299].

The application of “multi-omics” tools (genomics, proteomics, and others) and their perspectives are also significant in the advancement of microbial preservative applications in the food industry [300]. The power of omic sciences has enabled the rapid and effective search and screening of specific genes that encode antimicrobial peptides or lytic enzymes in phage or bacterial agents. This process was previously time-consuming and high-priced.

### 5.3. Nanotechnology

Combined with microbiology, nanotechnology has become a key ally in applying these bioproducts as preservatives in the food industry. It has been described how nano methods alone can contribute to the preservation of food, yielding promising results in other parts of the process, such as processing and packaging [301,302,303,304]. However, when combined with microbial biomolecules, these effects can be enhanced to extend shelf life and other aspects of the products, and they may even reinforce the concept of “green technology” in the production process, ultimately pleasing the final consumer.

A study on the production of organic nanodots from microalgae (*Spirulina platensis*) observed a promising role as a food preservative, as these nano-molecules inhibited the growth and biofilm formation of common pathogenic bacteria and fungi while also presenting better sensory quality results in the studied beverage [305]. A review paper discussed that bacteriocins produced by LAB have an antimicrobial properties range that is enhanced when conjugated with silver nanoparticles [306]. In another article, nanoparticles were discussed as conjugated agents to improve the action of essential oils and biopolymers in more effective food packaging [307]. It demonstrates the versatility and wide range of applications that nanotechnology can be used in the search for novel food preservative agents.

## 6. Microbial Fermenters in Postharvest Disease Management

Postharvest diseases cause significant losses in fruits and vegetables, resulting in annual losses of 10–50% in supply chains. These losses have serious consequences for food security and economic stability [308]. To manage postharvest diseases, synthetic chemical pesticides are commonly used. However, the emergence of multidrug resistance, changes in global climate, and the need for eco-friendly practices have led to an increasing use of microbial fermentates derived from fungi, including yeasts, and bacteria [309].

Microbial fermentates are biological control agents (BCAs) obtained from fermented broths or fractions that contain no viable cells. These fermentates include natural antibiotics, enzymes, organic acids, antimicrobial peptides, and various other microbial metabolites [310]. The benefits of using microbial fermentates include: (i) Non-toxic residues that comply with export and clean label requirements; (ii) Alignment with the circular bioeconomy through production from agro-industrial byproducts; and (iii) Multiple mechanisms of action that reduce the risk of resistance development [280,309].

Currently, it is crucial to align industrial production processes with the bioeconomy. In this context, several examples are found of the use of agro-residues in the production of microbial fermentates [311]. Table 8 shows examples of Microbial Fermentates in Postharvest Disease Control.

Cell-free supernatants of two strains of *Lactiplantibacillus plantarum*, isolated from citrus fruit and exhibiting antifungal activity, were obtained after fermentation in a medium based on lemon peel powder, a byproduct of agricultural waste. The supernatant exhibited antimicrobial action against *Penicillium digitatum* and *Penicillium italicum,* the causal agents of green and blue moulds, respectively. Additionally, it showed activity against *Alternaria alternata*, *Colletotrichum gloeosporioides*, *Camponotus karsti*, *Phytophthora citrophthora*, *and Phytophthora nicotianae* [280,281].

To prevent postharvest diseases, in some instances, microbial cells were used. A conidial suspension of *Aerobasidium pullulans* was applied directly to the plants, particularly for addressing White Haze on apples caused by *Entyloma belangeri*, *Golubevia pallescens*, and *Tilletiopsis washingtonensis* [312,313].

The commercial preparations BlossomProtect^®^, Grape Botector^®^, and BlossomProtect Grape^®^ Botector use Aerobasidium pullulans to prevent Pome Fruit and grapes from being affected by *Penicillium*, *Botrytis*, and *Monilinia*. Other examples include Nexy^®^, a postharvest technology that utilizes the naturally occurring yeast *Candida oleophila* strain to protect Citrus fruits, Bananas, and pome fruits. It is a broad-spectrum biofungicide active against several pathogens, including *Botrytis cinerea*, *Penicillium* spp., and *Colletotrichum musae*, with a preventive action [314,315].

**Table 8:** Representative Microbial Fermentates for Postharvest Disease Management.

Postharvest Disease	Etiologic Microorganism	Microbial Agent	Crop/Host	Reference
Green and blue molds	*Penicillium digitatum* and *P. Italicum*	Cell-free supernatants lactobacilli strains	Lemmon	[281]
Anthracnose	*Colletotrichum plurivorum*	kernel cake and pineapple peel fermented with *lactobacillus plantarum*	Mango	[314]
Gray mold	*Botrytis cinerea*	Bacillus fermentates/culture filtrates	Tomato, strawberry, grapes	[315]
Pepper rot	*Phytophthora capsici*	Fermentation supernatant of *Lactobacillus plantarum*	Pepper	[316]
Green mould	*Penicillium digitatum*	fermentates from *Candida peltata*	Citrus fruits	[317]
White rot	*Coniella diplodiella Coniella vitis*	metabolites of the *PaeniBacillus pisiformis* ZBSF and/or the *Paenibacillus pisiformis* ZB	Grapes	[318]

## 7. Bacterial Contaminants in Food

Microbial contamination of food is a significant public health concern, as it is responsible for numerous foodborne diseases. More than 250 causative agents have been identified worldwide, and studies indicate an increasing frequency of outbreaks [319]. Research has shown that foodborne illnesses commonly manifest through symptoms such as abdominal cramps, diarrhea, vomiting, chills, anxiety, and respiratory distress. These effects can result from direct microbial infection, as seen with *C. perfringens*, or from toxins produced by bacteria, such as those involved in *C. botulinum* poisoning. Certain foods, including poultry, processed meats, seafood, dairy, fruits, and vegetables, are frequently linked to these infections due to contamination during production, handling, or storage [320]. Therefore, foodborne illnesses are classified into two types: foodborne infections, where the pathogen establishes itself in the body and causes symptoms after an incubation period, and foodborne intoxications, which result from the ingestion of preformed toxins in food, leading to a faster onset of symptoms [321].

According to the WHO, food and water contamination leads to approximately two billion deaths annually from diarrheal diseases, with children under five years old accounting for 30% of these cases. In the United States alone, foodborne illnesses affect around 48 million people yearly, resulting in 128,000 hospitalizations and 3000 fatalities [322]. Bacteria are the leading agents of foodborne illnesses, exhibiting various shapes, classifications, and characteristics [321]. Among the primary pathogens responsible for these health risks are *Listeria monocytogenes*, *E. coli*, *Staphylococcus aureus*, *Salmonella* spp., *Clostridium* spp., and *Campylobacter* spp. [322].

*L. monocytogenes* is a Gram-positive bacterium responsible for listeriosis, a severe foodborne infection characterized by septicemia, meningitis, and maternal-fetal complications. The disease primarily affects immunocompromised individuals, the elderly, and pregnant women, with a high mortality rate ranging from 20% to 30%. Although less frequent than other foodborne infections, listeriosis is a significant public health concern due to its severity and potential for severe complications. Transmission occurs through the consumption of contaminated foods, such as unpasteurized dairy products and processed meats. Furthermore, a study demonstrated that this bacterium can grow at low temperatures, such as 8 °C, increasing the risk of infection even in refrigerated foods. The largest recorded outbreak occurred in South Africa, infecting 937 people and resulting in a 27% mortality rate [323,324].

*Salmonella* genus, belonging to the *Enterobacteriaceae* family, is taxonomically classified into two species: *S. enterica* and *S. bongori*. Most human infections are caused by *S. enterica*, a flagellated, Gram-negative, facultatively anaerobic bacillus responsible for many foodborne illnesses worldwide. *Salmonella* accounts for approximately 26% of bacterial foodborne infections in the United States, leading to an estimated 1.35 million cases, 26,500 hospitalizations, and 420 deaths annually. Clinically, salmonellosis manifests in various forms, ranging from enteric fevers (such as typhoid and paratyphoid) to acute gastroenteritis, bacteremia, and chronic carrier states. Transmission typically occurs through the consumption of food or water contaminated with fecal matter from infected humans or animals, as well as direct contact with infected animals. The primary sources of contamination include poultry, eggs, pork, beef, fruits, vegetables, and other produce [325].

*E. coli* is a diverse group of Gram-negative bacteria, with most strains harmless to humans. With over 700 distinct strains or serotypes found in nature, water, and various foods, *E. coli* can possess numerous virulence factors. These factors, often located on mobile genetic elements or plasmids, include endotoxins, exotoxins, and mechanisms for adhesion, invasion, and iron acquisition, which contribute to the pathogenicity of harmful strains [326]. Certain strains, such as Shiga toxin-producing *E. coli* (STEC), can cause severe foodborne illnesses. These strains produce Shiga toxins, which are responsible for symptoms ranging from abdominal cramps and bloody diarrhea to life-threatening complications like hemolytic uremic syndrome (HUS), which can lead to kidney failure. The primary sources of infection include consuming contaminated raw or undercooked meat, unpasteurized milk, and fresh produce that has not been properly washed. *E. coli* O157:H7 is the most significant STEC strain in terms of public health concerns. Cattle are the main reservoir of STEC, but other ruminants and environmental sources, such as contaminated water, also contribute to transmission. The increasing association of outbreaks with fresh fruits and vegetables highlights the importance of food safety measures. Preventive strategies include proper cooking, hygiene practices, and minimizing cross-contamination during food handling [327].

Early and accurate detection of bacterial contaminants is crucial for ensuring food safety and preventing disease outbreaks. Various analytical methods have been developed to identify and quantify these bacteria in food, enabling a swift response to contamination. The following section will explore the main techniques for detecting foodborne pathogens and ensuring public health.

Identifying foodborne pathogens involves various laboratory techniques, including conventional culture methods, immunological assays, molecular approaches, and emerging technologies such as biosensors [257]. The choice of method depends on the molecular targets and the sensitivity and specificity required for detecting the pathogens [328].

This section provides a concise overview of the primary methods employed by the scientific community to detect microbial contamination in food and identify the contaminating agents. It covers conventional culture-based techniques and rapid molecular approaches, emphasizing their advantages and limitations.

### 7.1. Conventional Bacteriological-Based Methods

Conventional methods for detecting foodborne pathogens are based on the morphological, physiological (phenotypic), genotypic, and biochemical characteristics of microorganisms. The approach used depends on the type of sample and the target organism. These methods mainly include traditional culture-based techniques and biochemical assays.

Culture-based methods are the oldest and most established techniques for identifying foodborne pathogens. They follow a step-by-step process involving pre-enrichment, selective and differential plating, confirmation, and strain identification. Pre-enrichment helps recover damaged cells, increase pathogen concentration, and rehydrate dried food samples, while selective enrichment promotes the growth of specific pathogens using targeted media [329].

These methods are most effective when the growth requirements of the target organisms are well-known. Culture media play a crucial role in enriching, isolating, and differentiating pathogens, facilitating the detection of *E. coli*, *L. monocytogenes*, *Salmonella*, *Campylobacter*, *Clostridium*, Enterobacteria, and *Bacillus* species [329]. Complementary techniques, such as MALDI-TOF, enhance the detection of foodborne fungal pathogens. Culturing sample extracts in cell lines and observing cytopathic effects can also identify foodborne viruses. Quantification is performed using plaque assays, tissue culture infectious dose 50 (TCID_50_), or the most probable number method [330].

Biochemical tests often support culture-based methods by using specific compounds that react with target pathogens while suppressing the growth of other organisms. These tests usually involve incubation in liquid or solid media. Standard biochemical assays include oxidase, catalase, indole, methyl red, Voges-Proskauer, and sugar fermentation tests, which help distinguish bacteria based on their metabolic responses [331,332]. Despite being cost-effective and easy to use, conventional methods are time-consuming, labor-intensive, and require skilled personnel. Complete identification can take up to seven days due to the multiple steps involved, such as pre-enrichment, incubation, and biochemical analysis [333,334].

### 7.2. Rapid Detection Techniques for Foodborne Pathogens

Fast, accurate, cost-effective, and easy-to-use methods are critical for detecting foodborne pathogens. One of the main advantages of such methods is that they allow food processors to obtain results before products leave the processing facilities, helping to prevent potential outbreaks of foodborne illnesses [334].

A biosensor is a tool used to detect a target pathogen by using biological sensing elements that produce a specific, measurable signal. A physical transducer then captures, enhances, processes, and interprets this signal [335]. Substances such as antibodies, enzymes, aptamers, antimicrobial peptides, bacteriophages, biomimetic materials, cells, tissues, and nucleic acid probes are considered biological sensing elements (bioreceptors) [333,336]. Biosensors commonly used to detect foodborne pathogens rapidly include optical and electrochemical biosensors [337].

Optical biosensors offer high sensitivity, enabling rapid detection and making them practical tools for identifying foodborne pathogens. They have been successfully applied in detecting *Salmonella* spp., *Escherichia coli*, and *S. typhimurium* [338,339,340]. Electrochemical biosensors function by using an electrode as a signal transducer. When the target analyte undergoes an electrochemical reaction at the electrode interface, it induces measurable changes in current, voltage, resistance, or conductivity, which the sensor detects [341]. This approach has been proven to detect bacterial pathogens, such as Salmonella, even at very low concentrations [342].

Biosensors that integrate microfluidic platforms with advanced imaging technologies, such as lensless microscopy and smartphone-based imaging, facilitate the real-time visualization and analysis of captured viral particles, holding significant potential for adaptation in the detection of foodborne pathogens [343].

Rapid or real-time detection provides immediate feedback on food products, enabling swift action to prevent consumption or further contamination [344]. However, biosensors may encounter challenges in complex environments and can have drawbacks, such as using enzymes as bioreceptors, which increases production costs and decreases shelf life [330].

### 7.3. Immunological Methods

Immunological detection of foodborne pathogens relies on the specific binding between antigens from the target microorganism and corresponding monoclonal or polyclonal antibodies [329]. Standard immunological methods include enzyme-linked immunosorbent assay (ELISA), immunomagnetic separation (IMS), agglutination assays, immunoprecipitation, immunochromatography, and lateral flow immunoassays [329]. Among these, ELISA is the most widely used for detecting foodborne pathogens [345]. ELISA detects antigen-antibody complexes through enzyme reactions, offering high sensitivity, specificity, rapid results, and the ability to test multiple samples with minimal labor. Common enzymes include alkaline phosphatase, horseradish peroxidase (HRP), and β-galactosidase [346]. ELISA formats include direct, indirect, sandwich, and competitive types, with the sandwich ELISA being the most specific [334].

This method has been successfully applied to detect *B. cereus*, *S. aureus*, *Salmonella*, and *E. coli* O157 [328,347,348,349]. Compared to conventional methods, immunoassays offer faster results; however, their performance may be compromised by antigen variability, limited sensitivity, and challenges in measuring enzyme activity.

### 7.4. Molecular-Based Methods

Molecular-based rapid methods utilize nucleic acid amplification to detect and quantify the DNA or RNA of specific pathogens. This involves hybridizing a synthetic oligonucleotide (probe or primer) with a complementary target sequence [333]. Among these techniques, polymerase chain reaction (PCR) is the most widely used [350].

PCR amplifies a specific DNA fragment through repeated cycles of denaturation, primer annealing, and extension using thermostable DNA polymerase. Each cycle doubles the target sequence, enabling highly sensitive detection [351]. It is currently the most effective tool in diagnosing foodborne pathogenic bacteria, viruses, and fungi [352]. Improved PCR methods, such as multiplex PCR, which amplifies multiple gene targets, can rapidly detect microorganisms of the same species or different species in a single reaction [353]. Other PCR-based assay methods, such as loop-mediated isothermal amplification (LAMP), have also demonstrated rapid detection of *Listeria monocytogenes* with high sensitivity [354].

Real-time or quantitative PCR (qPCR) is more widely used to detect foodborne bacterial pathogens in food samples than multiplex PCR because Multiplex PCR can face challenges with primer interactions leading to cross-reactivity, whereas qPCR minimizes this risk, enhancing accuracy [355,356]. *Salmonella*, *E. coli*, *Campylobacter*, and *L. monocytogenes* have been detected and quantified in cheese, chicken, beef burgers, turkey, pork, eggs, and fish using qPCR [357,358].

PCR-based techniques are faster, more sensitive, highly reproducible, and versatile compared to most culture-based and immunoassay methods, making them a preferred choice for pathogen detection [359,360]. The main drawback of amplification methods is that their high sensitivity increases the risk of cross-contamination, as they amplify tiny amounts of DNA millions of times [257,270,331,348].

### 7.5. Novel and Advanced Detection Methods

Advanced methods, such as mass spectrometry, have become essential tools for pathogen identification [361]. MALDI-TOF MS is increasingly applied for detecting foodborne pathogens, particularly those from key bacterial groups such as *E. coli*, *S. typhimurium*, *S. aureus*, *C. jejuni*, *Klebsiella*, and *L. monocytogenes* [362]. Refined techniques, such as coupling solid-phase microextraction (SPME) with comprehensive two-dimensional gas chromatography quadrupole time-of-flight mass spectrometry (GC×GC-QTOFMS), have also demonstrated effectiveness in detecting microbial volatile compounds associated with foodborne pathogens [363]. Mass spectrometry-based approaches, including MALDI-TOF MS, are rapid, accurate, cost-effective, and high-throughput, making them well-suited for detecting and monitoring pathogenic microorganisms [364]. However, the accuracy of identification can be affected by factors such as culture conditions and sample preparation [365].

The CRISPR-based detection method represents a significant breakthrough in the rapid identification of foodborne pathogens. This technique relies on two fundamental processes: adaptation and interference. These processes were developed based on the distinct genetic adaptation systems observed in archaea and bacteria, which are designed to defend against foreign genetic elements such as viruses and plasmids [366].

During the adaptation phase, segments of foreign DNA—known as protospacers—are incorporated into the CRISPR locus of the host genome, forming a genetic archive of past infections. In the interference phase, CRISPR-associated (Cas) proteins, guided by CRISPR RNA (crRNA), recognize and cleave complementary sequences in invading DNA, thereby neutralizing the threat [367].

The specificity of this system is determined by the crRNA, which can be engineered to target any desired nucleotide sequence. This method has been successfully adapted to detect specific nucleic acid sequences of foodborne pathogens with high accuracy. Unlike conventional methods, it allows for rapid and precise detection of pathogen genetic material in complex samples without the need for extensive preprocessing [366].

One notable application is the development of systems such as DETECTR (DNA Endonuclease-Targeted CRISPR Trans Reporter)—based on CRISPR/Cas12a—which have demonstrated high specificity and efficiency in detecting foodborne pathogens such as *Salmonella* in milk and chicken, as well as other pathogens like *Staphylococcus aureus* [368]. Other systems, such as SHERLOCK (Specific High-Sensitivity Enzymatic Reporter UnLOCKing)—based on CRISPR/Cas13—have also been employed for the detection of *Pseudomonas aeruginosa* and *Encephalomyocarditis virus* [369,370].

However, the technique also presents some limitations. It often depends on nucleic acid amplification methods such as PCR, which can increase the overall cost. Additionally, the nonspecific collateral cleavage activity of specific CRISPR-Cas systems may reduce detection specificity. The cost and stability requirements for enzyme production, processing, and storage also pose challenges [366].

In conclusion, detecting foodborne pathogens encompasses a broad spectrum of laboratory methodologies, ranging from conventional culture-based techniques and immunological assays to advanced molecular methods and emerging technologies such as biosensors and mass spectrometry. The selection of an appropriate detection strategy is contingent upon several factors, including the target pathogen, desired sensitivity and specificity, sample matrix, and overall cost-effectiveness. These approaches provide a robust framework for food safety surveillance, aiming to strike a balance between analytical precision, operational efficiency, and practical applicability.

## 8. Challenges, Perspectives, and Conclusions

Microorganisms provide a diverse toolkit of strategies that can collectively enhance the sustainability of food production, as demonstrated throughout this review. Microbial fermentations, including the emerging field of precision fermentation, can convert a wide range of substrates, including agro-industrial wastes, into nutrient-rich foods and ingredients. These processes provide alternatives to traditional animal products while reducing biowaste. Microbially derived additives such as enzymes, organic acids, bacteriocins, and natural pigments have proven effective in enhancing food safety and quality by reducing reliance on synthetic chemicals. Likewise, incorporating probiotics and prebiotics into foods enhances their nutritional functionality and provides health benefits for consumers. Taken together, these innovations demonstrate how microbiology can contribute to key sustainability goals, including waste valorization, extending shelf life, and advancing progress toward a circular food economy.

Despite these benefits, several challenges must be addressed to realize the full potential of microbial solutions in the food sector. A foremost issue is scale-up. Translating laboratory-scale successes to industrial-scale production is complex. Conditions that are easily controlled in small reactors become difficult to maintain uniformly in large bioreactors, which makes it challenging to sustain high yields and consistent product quality. Economic feasibility is another concern. The capital required to build and operate fermentation facilities is substantial, and operating costs for feedstocks, energy, and downstream processing can keep microbially derived products more expensive than conventional options in the short term. Achieving economies of scale and optimizing supply chains will be crucial to reducing costs and making these sustainable alternatives competitive.

Beyond scale and cost, important regulatory and technical hurdles remain. Navigating approval pathways for novel microbial foods and ingredients requires rigorous safety and nutritional assessments, with extensive evidence expected before new products reach the market. These processes can be time-consuming and expensive, particularly when they involve genetically modified microbes or unfamiliar classes of additives. Technical challenges also persist. Target pathogens may develop reduced susceptibility to biological preservatives; prolonged use of a single bacteriocin, for example, can select for resistant strains and diminish efficacy over time. This risk can be mitigated by combining multiple biopreservatives or using hurdle approaches. Continuous strain optimization is also necessary to improve yields and robustness; however, engineering consistently high-performing strains is not trivial and remains resource-intensive. Variability in substrates poses another obstacle: when processes rely on agricultural byproducts or food waste, inconsistent composition can drive fluctuations in efficiency and yield. Flexible bioprocesses and pretreatment methods will be necessary to handle this variability and support reliable operation at a large scale.

Looking ahead, research and development should focus on bridging these gaps through multidisciplinary approaches. More validation at a meaningful scale, including pilot plants and techno-economic analyses, is needed to demonstrate safety, efficacy, and true scalability in production environments. Improving the cost-effectiveness of bioprocessing is also a priority. This includes optimizing fermentation parameters to increase productivity, reducing energy and water consumption, and utilizing low-cost renewable substrates to lower expenses. Integrating advanced omics and synthetic biology can accelerate progress. High-throughput genomics and metabolomics can identify targets for strain improvement, while metabolic engineering enables the design of strains with higher yields or novel functionalities. Combined with sustainable bioprocess engineering, these tools allow the development of precision fermentation systems that maximize output while minimizing resource use. Notably, utilizing agricultural residues and food waste as feedstocks can reduce costs and promote sustainability by converting waste streams into valuable inputs, aligning with the circular bioeconomy.

From a broader perspective, microbial applications are poised to transform food safety, nutrition, and environmental stewardship. By decreasing reliance on chemical additives and resource-intensive animal agriculture, microbial solutions can reduce the ecological footprint of food production while protecting public health. These biotechnologies also contribute to a circular bioeconomy by valorizing waste and enabling more efficient use of land and water than many conventional systems. To capture these benefits, continued scientific innovation must proceed alongside supportive policy and regulatory alignment, with clear guidelines that ensure safety without slowing progress. Equally important is collaboration across disciplines and sectors. Microbiologists, food engineers, industry partners, and regulators should collaborate to address scaling challenges and foster public trust through transparent communication about foods derived from microbial processes. With sustained investment and careful oversight, microbial innovations can help transform the global food system into one that is safer, more resilient, and more environmentally sustainable. Table 9 synthetically categorizes microbial applications and identifies the main research trends.

Overcoming the current challenges will unlock the full potential of microbes in sustainable food production and pave the way for a more circular and secure food future.

## Figures and Tables

**Table 1:** Comparative Analysis of Submerged Fermentation (SmF) and Solid-state Fermentation (SSF).

Parameter	SmF	SSF
Substrate type	Soluble substrates in liquid media	Solid substrates with minimal or no free water, often using agro-industrial residues
Microbial growth preferences	Ideal for bacteria and yeast that thrive in liquid environments	Suited for filamentous fungi and certain bacteria adapted to low-moisture conditions
Water activity (aw)	High water activity	Low water activity
Aeration and oxygen transfer	Oxygen transfer due to agitation and aeration systems	Relies on diffusion
Energy consumption	Higher energy requirements for agitation, aeration, and temperature control	Lower energy demands often passive processes.
Contamination risk	Elevated risk due to high moisture and nutrient availability	Reduced risk owing to low moisture
Product yield and concentration	Often lower due to dilution in the aqueous phase	Higher concentration of products; easier downstream processing
Fermentation time	Typically shorter due to rapid growth in liquid	Longer due to slower metabolism in solid substrates
Process monitoring and control	Real-time control of pH, temperature, and others	Difficult to monitor and control accurately
Scale-up and industrial application	Well-established with standardized equipment	Challenging due to complexity and heterogeneity
Environmental impact	High wastewater and energy use	Minimal wastewater and resource use; sustainable
Economic considerations	Higher operational costs	Cost-effective with low-input substrates
Technological maturity	Mature and widely adopted	Emerging; increasing industrial interest

**Table 3:** Food additives with function, food examples, and potential harm to health.

Additive	Function	Common Foods	Potential Health Impact	References
Monosodium glutamate (MSG)	Flavor enhancer	Instant noodles, processed meats	May cause headaches, nausea (in sensitive individuals)	[89]
Sodium nitrite	Preservative, color fixative	Cured meats (bacon, ham)	May form carcinogenic nitrosamines	[90]
BHA (Butylated hydroxyanisole)	Antioxidant, preservative	Chips, cereals, butter	Possible endocrine disruptor linked to cancer in high doses	[91]
Potassium bromate	Dough conditioner	Bread, baked goods	Possible carcinogen, banned in some countries	[92]
Tartrazine (E102)	Coloring agent	Soft drinks, candies	Hyperactivity, allergic reactions	[93]
Sodium benzoate	Preservative	Sodas, sauces	Trigger allergies, potential carcinogens	[94]
Carrageenan	Thickener	Dairy products	Linked to gastrointestinal inflammation	[95]
Aspartame	Sweetener	Diet sodas, gums	Headaches, metabolic disturbances	[96]
Titanium dioxide (E171)	Coloring agent	Candies, chewing gum	Potential genotoxic effects, banned in some regions	[97]
Polysorbate 80	Emulsifier	Ice cream, salad dressings	Disrupt gut bacteria linked to inflammation	[98]
Sorbitol	Sweetener, humectant	Sugar-free gum, candies	Causes bloating, laxative effects	[99]
Sucralose	Artificial sweetener	Diet sodas, sugar-free products	Alter gut microbiota, potential metabolic effects, and cardiovascular disease	[100,101]

**Table 4:** Comparison of International Regulatory Frameworks for Microbial Food Additives.

Agency	Region	Key Lists/Frameworks	Safety Requirements	References
Food and Drug Administration (FDA)	United States	GRAS—Generally Recognized as Safe	Toxicological safety, history of safe use, scientific consensus on safety for intended use	[106,107,108]
European Food Safety Authority (EFSA)	European Union	QPS—Qualified Presumption of Safety	Non-pathogenicity, absence of toxigenicity, genetic stability, absence of antimicrobial resistance genes, allergenicity assessment	[32,109]
Agência Nacional de Vigilância Sanitária (ANVISA)	Brazil	RDC Resolutions (e.g., RDC 728/2022)—Official list of approved microbial strains	Non-pathogenic, non-toxigenic, genetically stable strains, and industrial performance consistency	[110,111,112]
China Food and Drug Administration (CFDA, currently reorganized under the State Administration for Market Regulation (SAMR) and the National Health Commission (NHC).	China	National Food Safety Standards (GB standards, NFSSs)	Toxicological evaluation, allergenicity testing, and safety under intended conditions of use	[113,114,115,116]

**Table 5:** Microbial enzymes applied in the food industry.

Enzyme	Microbial Sources	Main Function	Industrial Applications	References
Peptidases	*A. oryzae*, *A. niger*, *Rhizopus* spp., *B. subtilis*, *B. licheniformis*	Hydrolysis of peptide bonds; milk coagulation	Cheese production (chymosin/rennet), whey hydrolysis, dough extensibility in baking	[105,125,126,127,128,129,130]
Cellulases and Pectinases	*T. reesei*, *A. niger*	Degradation of cellulose and pectin; macerating action	Juice extraction and clarification, viscosity reduction, pulp liquefaction	[131,132,133,134,135,136,137,138,139,140]
Xylanases	*A. niger*, *A. oryzae*, *T. reesei*, *B. subtilis*	Hydrolysis of xylan; improvement of rheological properties	Increased bread loaf volume and texture, clarification of juices and beer	[140,141,142,143,144,145,146]
α-Amylase and Glucoamylase	*A. niger*, *R. oryzae B. subtilis*, *B. amyloliquefaciens*	Conversion of starch into fermentable sugars	Bread making, syrup production, alcoholic beverages	[147,148,149,150,151,152]
Lactase (β-galactosidase)	*K. lactis*, *A. oryzae*, *B. circulans*	Hydrolysis of lactose into glucose and galactose	Low-lactose dairy products, prevention of crystallization in ice creams, production of prebiotic GOS	[153,154,155,156,157,158]
Lipases and Phospholipases	*T. lanuginosus*, *R. miehei*, *C. rugosa*, *A. oryzae*	Hydrolysis and modification of lipids	Cheese flavor development, bakery emulsification, oil refining, structured lipids	[159,160,161,162,163,164,165]
Laccase	*Trametes versicolor*, *Pleurotus ostreatus*, *A. oryzae*	Oxidizes phenolic compounds; contributes to beverage stabilization	Juice, wine, and beer clarification improve dough structure in baking	[159,166,167]
Naringinase	*A. niger*, *Penicillium decumbens*, *R. nigricans*	Hydrolyzes naringin (a bitter flavonoid)	Reduction of bitterness in citrus juices (e.g., grapefruit)	[164]
Tannase	*A. niger*, *A. oryzae*	Removes polyphenolic compounds; reduces astringency	Brewing industry and instant tea production	[165,168]
Catalase	*Micrococcus luteus*, *A. niger*, *B. subtilis*	Decomposes hydrogen peroxide into water and oxygen	Preserves dairy and packaged foods; extends shelf life and improves microbiological safety	[169]
Phytase	*A. niger*, *E. coli*, *B. amyloliquefaciens*	Hydrolyzes phytic acid; releases bioavailable minerals	Animal feed supplementation; enhances mineral absorption, reduces environmental phosphorus output	[170]
Transglutaminase	*Streptoverticillium mobaraense*, *Streptomyces mobaraense*, *B. subtilis*, *B. sphaericus*	Catalyzes covalent cross-links between glutamine and lysine residues in proteins	Improves the texture and elasticity of processed meats, dairy products, and baked goods	[171,172]
Glucose oxidase	*A. niger*, *A. oryzae*, *P. amagasakiense*	Oxidizes glucose to gluconic acid and hydrogen peroxide	Improves dough structure and bread volume; reduces glucose in functional beverages	[173,174]
Pullulanase	*B. acidopullulyticus*, *B. subtilis*, *B. deramificans*, *Klebsiella* spp.	Hydrolyzes α-1,6-glycosidic bonds in pullulan and branched starches	Used in starch processing to produce glucose and maltose; enhances saccharification in brewing and syrup production	[175,176]

**Table 6:** Microbial Organic Acids in Foods: Producers, Functions, and Industrial Applications.

Organic Acid	Main Microbial Producers	Functions in Food Products	Industrial Application	References
Acetic acid	*Acetobacter* spp., *Gluconacetobacter* spp., *Gluconobacter* spp.	Flavoring, preservative, functional bioactivity in fermented foods	Vinegar, kombucha, cocoa, sour beer	[177,178,179]
Citric acid	*Aspergillus niger*, *Trichoderma reesei*, *Yarrowia lipolytica*	Flavoring, acidity regulator, preservative; component in bioactive food packaging	Soft drinks, effervescent salts, medicinal citrates, biofilm-based packaging	[180,181,182,183,184,185,186]
Lactic acid	Lactic acid bacteria (*Lactobacillus* spp., *Lactococcus* spp.), *Rhizopus oryzae*, *Mucor* spp.	Flavoring, acidity regulator, preservative; antimicrobial activity enhancer	Bakery, jams, candies, beverages, bioplastics/packaging	[187,188,189,190]
Propionic acid	*Acidipropionibacterium* spp., *Propionibacterium* spp.	Flavoring, preservative in bakery and dairy; nutritional co-products (vitamins)	Bread, cheese, jams, fermented foods	[191,192,193,194]
Succinic acid	*Actinobacillus succinogenes*, *Mannheimia succiniciproducens*, *Basfia succiniciproducens*	Flavoring, preservative, bread softener; precursor for biochemicals	Bread, beverages, biochemical platform for plastics and solvents (engineered yeasts)	[195,196,197,198,199,200,201]

**Table 7:** Representative microbial bioproducts applied in the food industry, highlighting their primary functions and industrial applications.

Additive Type	Compound/Example	Functions in Food Products	Industrial Applications	Main Microbial Producers	References
Texturizers and Stabilizers	Xanthan gum	Thickener, stabilizer, emulsifier	Low-fat and gluten-free formulations, sauces, dressings	*Xanthomonas campestris*	[208,209]
	Pullulan	Thickener, film-former	Edible films, coatings, candies	*Aureobasidium pullulans*	[210]
	Dextran	Stabilizer, prevents crystallization	Confectionery, ice cream, bakery	*Leuconostoc* spp., *Lactobacillus* spp.	[208]
	Gellan gum	Gelling and stabilizing agent	Beverages, jams, dairy, fruit coatings	*Pseudomonas elodea*	[211]
	Bacterial cellulose	Texture, water retention, fat replacement	Ice cream, tofu gels, meat products	*Gluconacetobacter* spp.	[208]
Colorants	Carotenoids (β-carotene, astaxanthin)	Natural pigments, antioxidant	Dairy, beverages, nutraceuticals	*Blakeslea trispora*, *Dunaliella salina*, *Haematococcus pluvialis*	[202,212]
	Monascus pigments	Natural red pigments	Fermented foods, sauces, beverages	*Monascus* spp.	[213]
	Phycocyanin, chlorophylls	Natural pigments, antioxidant	Confectionery, beverages, jellies	*Arthrospira* sp. (Spirulina), microalgae	[207]
Sweeteners	Xylitol	Natural sweetener, low-calorie	Sugar-free confectionery, chewing gum	*Cyberlindnera dasilvae* sp. nov., engineered *S. cerevisiae*	[214]
	Sorbitol, mannitol	Sweeteners, humectants	Bakery, sugar-free foods, pharmaceuticals	*Zymomonas mobilis*, *Lactobacillus plantarum*	[215,216]
	2′-Fucosyllactose (2′-FL)	Functional sweetener, prebiotic	Infant formula, functional foods	Engineered *E. coli*, *B. subtilis*	[203]
Flavorings and Aroma	Esters, aldehydes, ketones (e.g., ethyl acetate, benzaldehyde|)	Flavor enhancers	Fermented foods, beverages	*Lactobacillus* spp., *Acetobacter* spp., *Penicillium* spp.	[204]
	Vanillin (biovanillin)	Aroma compound	Beverages, dairy, bakery, chocolate	*Aspergillus niger*, *Penicillium cinnabarinum*	[206]
	Fruity aromas (e.g., ethyl lactate)	Flavor enhancers	Fruit juices, fermented products	*Ceratocystis fimbriata*, yeasts, LAB	[205,206]
Functional and Nutritional Bioproducts	β-glucans (paramylon, selacan)	Gelling, thickening, prebiotic	Functional foods, supplements	*Euglena* spp., *Agrobacterium* spp.	[217,218]
	Polyunsaturated Fatty Acids (PUFA) and vitamins	Nutritional enrichment	Beverages, pasta, baked goods	*Arthrospira* spp., *Chlorella* spp., *Nannochloropsis* spp.	[207]

**Table 9:** Synthesis of microorganism applications and current trends.

Category	Key Microbial Products	Applications in Food Systems	Emerging Research Trends
Fermentation & Biotransformation	Lactic acid, ethanol, organic acids, alternative proteins	Protein-rich ingredients, improved digestibility, waste valorization	Precision fermentation, strain engineering, CRISPR-enabled production
Microbial Food Additives	Bacteriocins, microbial enzymes, bio-pigments, Exopolysaccharide—(pullulan, xanthan)	Clean-label food enhancement, texture and color agents, health-promoting properties	Use of GRAS strains for multifunctional roles, metabolite optimization
Biopreservation & Safety	Antimicrobial peptides, reuterin, propionic acid, biosurfactants	Shelf-life extension, pathogen inhibition, reduced need for chemical preservatives	Natural antimicrobial cocktails, synergistic preservation systems
Sustainable Packaging	Edible films and coatings using microbial polymers (e.g., pullulan, gellan gum)	Biodegradable alternatives to plastic, smart coatings for perishables	Integration with nanotechnology, biodegradable multi-layered systems
Contamination Control & Detection	Biosensors, microbial indicators, diagnostic enzyme systems	Rapid detection of foodborne pathogens, smart packaging, contamination tracing	Point-of-need detection, AI-linked diagnostics, probiotic biosensors

## Data Availability

No new data were created or analyzed in this study. Data sharing is not applicable to this article.
